# Self-regulation training improves stress resilience in elite pre-pubescent female gymnasts

**DOI:** 10.3389/fpsyg.2024.1341437

**Published:** 2024-04-24

**Authors:** Giorgia Proietti, Milos Borozan, Amine Chaigneau, Loreta Cannito, Riccardo Palumbo, Regis Thouvarecq, Pierpaolo Iodice

**Affiliations:** ^1^University of Rouen Normandie, CETAPS UR 3832, Rouen, France; ^2^Department of Neuroscience, Imaging and Clinical Sciences, University “G. d’ Annunzio” of Chieti-Pescara, Chieti, Italy; ^3^Center for Advanced Studies and Technologies “CAST”, University “G. d’ Annunzio” of Chieti-Pescara, Chieti, Italy; ^4^Department of Human Studies, University of Foggia, Foggia, Italy; ^5^Movement Interactions Performance – MIP, Le Mans Université, Le Mans, France

**Keywords:** self-regulation, stress management, stress resilience, interoceptive awareness, biofeedback, elite athletes

## Abstract

**Introduction:**

In the context of young female athletes, namely elite gymnasts, effective stress management strategies not only enhance performance, but also reduce the risk of injuries and promote overall well-being. This study aims to investigate the effects of biofeedback-based training on stress management in prepubescent elite female gymnasts, recognizing its pivotal role in promoting healthy growth and proper training load management.

**Methods:**

Eight elite young female athletes from a top flight French national league club participated in an experimental condition involving four-week biofeedback training program to improve self-regulation skills, during both rest and stress phases. Additionally, each subject experienced a control condition, with entailed exposure to domain-specific motivational videos. Comprehensive evaluations of physiological parameters were conducted to assess the impact of biofeedback training, both before and after the training, as well as during the stress and recovery phases. Furthermore, an interoceptive body awareness test, using the MAIA questionnaire, was performed.

**Results:**

The results highlight a significant enhancement of the self-regulatory skills of the gymnasts in managing the selected physiological parameters—peripheral temperature (*p* < 0.05) and blood volume pressure (*p* < 0.05)—after the biofeedback treatment. Moreover, psychological data from the MAIA questionnaire revealed a noteworthy increase in interoceptive awareness (*p* < 0.001), particularly in the subscales of Not Distracting (*p* < 0.001), Attention regulation (*p* < 0.05), Emotional awareness (*p* < 0.05), and Self-regulation (*p* < 0.05).

**Discussion:**

Thus, we conclude that biofeedback training improves self-regulatory and psychological resilience under stressful conditions, while reducing sensitivity to gymnastics-specific stress.

## 1 Introduction

The world of high-level sports is characterised by intensive training, demanding competition, and public display of skills. These experiences can be overwhelming for the athletes, and there is evidence of negative long-term effects of the resulting stress on both their well-being and performances across the age envelope (Arnold and Fletcher, 2021). Excessive stress and tension represent a common experience for athletes in different sports and environments at all competition levels, from beginner to professional ones (Morgan, 1974; Greenspan and Feltz, 1989; Dugdale et al., 2002; Fletcher and Hanton, 2003). Stress-induced processes unfold over multiple aspects of individual functioning, consuming metabolic and attentional resources (Cañal-Bruland et al., 2010; Oudejans et al., 2011; Vine et al., 2016), and resulting in reduced enjoyment and a greater likelihood of injury (Nippert and Smith, 2008). Furthermore, stress represents a threat for the athlete’s ability to meet or exceed their performance goals (Davis and Sime, 2005) and subsequently leads to higher dropout rates (Dupee et al., 2016; Smith and Pollak, 2020). It is therefore not surprising that there is a growing body of research probing into the nature, determinants and impact of stress-related phenomena in sport. At the same time—drawing on both psychological (Jones, 2003; Balk and Englert, 2020) and technological (Dupee and Werthner, 2011) advances—researchers are exploring various stress-regulation techniques and interventions to help athletes deal with such demands and achieve more.

These endeavours rest upon the idea of maintaining the state of purposeful internal equilibrium in different conditions, referred to as psychophysiological self-regulation (Prinzel et al., 2001; Porges, 2007; Inzlicht et al., 2021). Research has shown that such a state is achieved through an efficient mind–body regulation, underlined by an adaptive interplay between sympathetic and parasympathetic nervous subsystems (Benarroch et al., 1995; Porges, 2007; Thayer and Lane, 2009). Recently, some authors have indicated that the efficiency of self-regulation is largely dependent on resilience, a key psychobiological capacity for maintaining normal functioning and health that involves adaptability to adversity (Fletcher and Hanton, 2003; Russo et al., 2012; Iodice et al., 2022). This theoretical framework is based on the neurovisceral integration model, which synthesizes extensive evidence linking the autonomic and central nervous systems in a functional and structural network involved in the emotional regulation of behaviour (Dworkin et al., 1994; Porges, 2007; Thayer and Lane, 2009; Pezzulo et al., 2018; Iodice et al., 2019; Yu et al., 2021). Particularly, stress can alter the flexibility of the circuitry underlying these delicate equilibria and thus lead to the suboptimal individual functioning (Marin et al., 2011; Mariotti, 2015; Anderson et al., 2019), with reduced degrees-of-freedom in the interactions with the environment (Damasio, 1998). Thus, given the ubiquity of the stress response and its documented negative impact on both short- and long-term health outcomes, as well as a variety of individual capacities (Thoits, 2010; O’Connor et al., 2021) and performances (Anderson et al., 2019; Lochbaum et al., 2022), an efficient stress management through self-regulation represents a highly desirable skill.

One of the most promising techniques for this purpose is biofeedback, a mind–body intervention that has the advantage of being unobtrusive, passive, and continuous (Brown, 1977; Sandweiss and Wolf, 1985; Bar-Eli et al., 2002; Dupee and Werthner, 2011; Strack et al., 2011). Unlike other stress-management techniques (for a review see Lehrer et al., 2000; Rumbold et al., 2012) biofeedback externalizes an individual’s physiological state, and allows the user to monitor changes in real time (Blumenstein et al., 1995; Schwartz and Andrasik, 2017; Kennedy and Parker, 2019). While many attempts to define biofeedback can be found in the scientific literature (for a review, see Peper and Shaffer, 2010; Schwartz and Andrasik, 2017; Schwartz et al., 2017), the core idea is simple: feedback is crucial for any kind of learning. By analogy with dancers practicing their craft in front of a mirror, biofeedback can be conceived as “a psycho-physiological mirror” (Peper and Schmid, 1983) which helps practicing and improving self-regulation (Dupee et al., 2016). Biofeedback instruments provide information about physiological processes that are normally beyond conscious access (such as cardiac rhythm, muscle or brain activity) in the form of auditory, visual or sensory signals (Peek, 2017). This information (i.e., feedback) helps to learn psychoregulatory control “for the purpose of improving health and performance” (Peek, 2017). Therefore, it can be said that individuals use biofeedback to create awareness of internal processes that are typically not consciously controlled (Landers, 1985; Zaichkowsky and Fuchs, 1988; Blumenstein and Weinstein, 2011). Once provided with feedback on physiological processes (in the form of heart rate or respiration rate), an individual can begin to formulate strategies for self-regulation.

The idea that self-regulation and/or resilience can indeed be enhanced through biofeedback training has received empirical support for both athletic (Rusciano et al., 2017) and non-athletic (Iodice et al., 2022) populations. In the case of the former, beneficial effects have been found for athletes in different sports, both individual (Bar-Eli et al., 2002; Galloway, 2011) and collective (Paul et al., 2012; Rusciano et al., 2017). Specifically, one of disciplines where the results of such practices have been explored is gymnastics. Ever since the early studies of Duda and Gano-Overway (1996a,b) highlighting the stress loads experienced by gymnasts, the need for effective stress-management interventions have only reverbed by recent high-profile cases of gymnasts such as Simone Biles withdrawing from the competitions due to excessive stress toil. To that aim, Codonhato et al. (2018) investigated the relationship between resilience and stress tolerance in elite-level gymnasts. The authors show that resilience has indeed a direct impact on stress management and injury prevention in adult athletes. However, with the lowering age of access to professional competition, athletes are being exposed to stress ever sooner in their developmental trajectories, which could potentially have a snowball effect later, both in terms of sport performance and life experiences (Jurimae and Jurimae, 2001; Caine and Nassar, 2005; White and Bennie, 2015; Smith and Pollak, 2020). Namely, acquiring psychophysiological self-regulation competencies during childhood is a key indicator for subsequent psychological well-being, the adoption of a healthy lifestyle, adaptive interpersonal behaviours, and overall mental health (Tangney et al., 2004; Moffitt et al., 2011; Howard and Williams, 2018; Robson et al., 2020).

Thus, the present study aims to explore the effects of four-week biofeedback training on elite prepubescent gymnasts, with a particular focus on enhancing their ability to cope with specific stressful situations that are most likely to induce harmful overarousal. The choice of this population addresses the notable scarcity of research on high-performance female athletes. Consequently, this gender-specific approach—particularly considering its unique hormonal and physiological factors—aims to contribute to a more balanced understanding of gender dynamics in high-performance sports. Namely, we aim to explore the possibility of using this training method in elite sportswomen to prevent physical injuries and early talent drop-out in emotionally demanding sports such as gymnastics. Specifically, a research protocol and a training program were developed, informed by prior scholarship (McHugh et al., 2010) and the inherent specificity of the target population. The training methods were adapted to the age profile of the participants. Informed by the prior literature on the subject, two physiological parameters (i.e., skin conductance level and peripheral skin temperature) were chosen as biofeedback training modalities. Both the electrodermal (skin conductance) and thermal (peripheral skin temperature) biofeedback have been found as suitable modalities for a stress-regulation intervention (Shaffer et al., 2016; Schwartz and Andrasik, 2017; Candia-Rivera et al., 2022). The stress response was elicited in a standardised setting and both the profile and the intensity of the participants’ stress response were gauged across different physiological parameters (Yu et al., 2018; Slavikova et al., 2020). Additionally, the levels of interoceptive awareness (Mehling et al., 2012) were evaluated also, aiming for a comprehensive assessment of the individual functioning following the biofeedback training.

The results of our study will help clarify the mechanisms underlying the training of self-regulation and thus resilience skills in young women. Finally, the possibility of incorporating this method into the training toolkit of young elite athletes will be discussed. We have delineated a research paradigm with the aim of probing if a biofeedback training could be an effective method for the development of robust stress-management abilities in young female athletes.

## 2 Materials and methods

### 2.1 Participants

A sample including 8 elite young female athletes from a top-flight French national league club was enrolled for the study (Table 1). Recruitment process involved a screening phase wherein a female investigator (GP) assessed the attainment of menarche by querying both the subjects and their respective parents or guardians. The athletes who had not yet reached menarche were considered pre-pubescent (Granados et al., 2015; Gillen et al., 2021).^1^ The participants compete in the high-level national tournaments and spend an average of 24 h of training per week.

**Table 1:** Anthropometric and athletic summary of the sample.

Age (years)	Experience (years)	Height (cm)	Body weight (kg)
10.9 ± 1.13	7.0 ± 1.20	139.3 ± 5.85	33.9 ± 5.64

None of the participants had any experiences with stress management interventions or mental preparation practices including meditation or yoga. Furthermore, none of them had musculoskeletal injuries nor took any anti-inflammatory drugs or corticosteroids for the duration of the study. After the data collection, the personal information of the participants was removed and thus the data analyses were completely anonymous. The study was carried out according to the ethical principles put forward in the Declaration of Helsinki and its subsequent amendments and it was approved by the Ethical Committee of the University of Rouen (2020-04-A).

### 2.2 Study design

We implemented a within-subject, cross-over design (Quintana and Heathers, 2014; Laborde et al., 2017). This choice was dictated by the exceptional nature of the study population (i.e., elite gymnasts), since it proved difficult to access this population for a continuous period in the same facility. In addition, all gymnasts performed the same number of training sessions with the same instructor. The study design comprised two phases (hereafter *conditions*): (i) Control condition (CTRL) which comprised 8 sessions, two per week, for a duration of 4 weeks and (ii) Experimental condition (with Biofeedback treatment—BF) which comprised 8 sessions, two per week, for a duration of 4 weeks. A two-week break (from the date of the CTRL condition post-test assessment) was provided between the two conditions.

**Figure 1:**
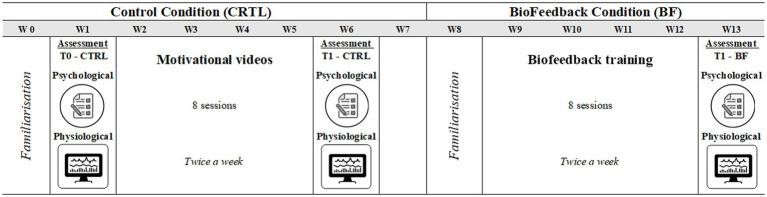
Weekly (W) timeline of the study setup. W0 and W8: familiarization; W1 and W6: assessment of the control condition; W13: evaluation of the biofeedback condition; W7: rest.

Familiarization sessions were held one week before both conditions with all participants. During these sessions, participants were introduced to the study objectives, the functioning and the purpose of the biofeedback, as well as psychological instruments that would have been used. Moreover, a detailed overview of session scheduling and pre-session prerequisites such as hydration and fasting were provided. Finally, participants had the chance to experience changes in physiological parameters through live demonstrations of the biofeedback equipment and sensors. Self-regulatory abilities of all participants were evaluated in three laboratory sessions, conducted during week 1 (before the start of CTRL condition), week 6 (after the end of CTRL condition), and week 13 (after the end of the BF condition) (Figure 1).

All testing was conducted in the same room, located at the Club headquarters, and never used by the participants in any capacity prior or during the experiment. Assessments encompassed both psychological and physiological components of stress experiences. Room temperature and humidity were kept constant during the evaluations. All sessions were conducted with the same artificial light to avoid possible influences of seasonal changes. To a feasible extent, every physiological recording was scheduled on an empty stomach.

Each assessment session lasted approximately 60 min and was structured as follows: after being welcomed by the experimenter, participants were invited for a psychological evaluation via the self-administered Multidimensional Assessment of Interoceptive Awareness (MAIA) questionnaire (Mehling et al., 2012). After a 5-min break, participants were asked to get themselves comfortable in an ergonomic chair and relax for some minutes, while the operator positioned the sensors. Afterwards, the standardised physiological assessment was performed.

#### 2.2.1 Psychological assessment

To assess the effects of biofeedback training on perceived individual self-regulation, the validated French version (Willem et al., 2022) of MAIA test was used. It is a self-report psychological instrument, aimed at measuring the efficacy of mind–body therapies and “capture the potential changes in body awareness over time as people learn and practice therapies that claim to enhance body awareness” (Mehling et al., 2012), such as meditation and biofeedback. It is composed of 32 items unfolding over 8 scales: *Noticing* (the ability to be aware of body sensations), *Not Distracting* (the tendency not to use distraction to cope with discomfort), *Not Worrying* (the tendency not to experience emotional distress), *Attention regulation* (the capacity to maintain and focus attention to body sensations), *Emotional Awareness* (the ability to attribute specific physical sensations to physiological manifestations of emotions), *Self-regulation* (assessing the ability to regulate distress by attention to body sensations), *Body Listening* (the tendency to actively listen to the body for insight) and *Trusting* (body perception as safe and trustworthy place) (Mehling et al., 2012). It encompasses different aspects of interoceptive body awareness, a key precursor of self-regulation capabilities and it presents satisfactory psychometric properties (Willem et al., 2022).

#### 2.2.2 Physiological assessment

It was carried out using a Sue Wilson profile “Optimizing performance and health suite” (Wilson, 2006), a standardised suite of 6 different stress tasks (Stroop Colour test, Math test, React Track Game, Dual Tracking Game, Anticipation and Brief Stressor) (Mehling et al., 2012), via ProComp Infiniti™ T7500M Biofeedback System manufactured by Thought Technology (Montreal, Quebec, Canada), with a BioGraph Infiniti™ software (version 6.0). The recorded signals included peripheral temperature (T, a sensor placed on the middle finger of the non-dominant hand) (Shaffer et al., 2016), skin conductance level (SCL, two separate sensors, placed on the index and pinky fingers of the non-dominant hand) (Boucsein, 2012; Peek, 2017), muscle activity (EMG electrode positioned on the right trapezius) (Arena et al., 1995), blood volume pressure (BVP, via a sensor placed on the index finger of the non-dominant hand) (Peper et al., 2006) and respiration rate (RR, strain-gauged belt, placed around the midsection of the abdomen and installed in an upright position during a maximal inhalation) (Ferguson et al., 2020).

It was performed during a standardised battery of stress-inducing tasks, presented on a computer monitor. The evaluation started with an establishment of a baseline physiological profile. Particularly, participants’ parameters were recorded during a task-free resting period of 4 min, of which 2 min with eyes closed, and then 2 min with eyes open and fixating a motionless cross on the computer monitor. Subsequently, each participant faced six cognitive stress tasks, lasting between 1 and 2 min, intermitted by recovery phases lasting 1 and a half minutes after each of the tasks. The physiological parameters were measured across all phases. The experimenter was always present, providing guidance and instructions for each task.

### 2.3 Training program

The entire experimental protocol was carried out at the Club Elan Gymnique Rouennais (Rouen, France) in a quiet and isolated office in the club’s facilities. Both study conditions included 8 sessions (two per week) each lasting 40 min.

In the *control condition* (CTRL), subjects were exposed to 40-min motivational videos featuring exceptional artistic gymnastics performances from the recent Olympic games and World Championships, accompanied by music (the videos were taken from the 2016 Olympic Games in Rio de Janeiro, 2018 World Championship in Doha and 2019 World Championships in Stuttgart). The experimenter (GP) followed each individual session via videoconference.

The *experimental condition* (BF) involved biofeedback training. Training sessions lasted around 40 min and included an initial 5-min relaxation phase followed by 20 min of biofeedback training. Each session was concluded with a brief follow-up on the subjective experience of the participant. Participants sat in an ergonomic chair and the training procedures were administered via a 15-inch computer monitor. The experimenter carefully provided detailed descriptions of each variable shown on the biofeedback training screen (i.e., skin conductance level and peripheral temperature) (Perry et al., 2011; Blumenstein and Hung, 2014). The control of the respiration rate and the diaphragmatic breathing were introduced as potential means of establishing control over physiological functions (Perry et al., 2011; Blumenstein and Hung, 2014; Zaccaro et al., 2018; Ely et al., 2020) and voluntary regulation of internal bodily states (Zaccaro et al., 2018). In the first session visual feedback was set in the form of a respiration pacer for each participant (e.g., 6 breaths/min) (Lehrer et al., 2000, 2013) and participants were instructed for natural and shallow abdominal breathing in accordance to their resonance frequency. The idea of balloon imagery (i.e., trying to fill the balloon with each inhale and deflate the balloon with each exhale) was introduced to participants to facilitate abdominal breathing and learn diaphragmatic breathing as well as minimizing thoracic movement, skills considered important in the quest to maintain control over physiological functions (Perry et al., 2011; Khazan, 2013; Laborde et al., 2017).

From the second session onwards, five biofeedback activities followed with a decreasing presence of feedback—skin conductance level training with visual and audio feedback, peripheral temperature training with visual and audio feedback, skin conductance level training with only visual feedback, peripheral temperature training with only visual feedback, skin conductance level training with only audio feedback, peripheral temperature training with only audio feedback (Iodice et al., 2022), and both with only visual feedback (in a form of value graph). Visual feedback was presented via a dynamic on-screen animation, while the audio feedback was provided as a sound which would become harmonious in proportion to the subject’s ability to follow the instructions. Participants were trained to decrease the skin conductance level or increase the peripheral temperature alternatively, using the provided feedback (Rusciano et al., 2017). The sessions included the equivalent, 10-min parts of thermal and electrodermal feedback, presented in a randomised order. At the end of each session, the data and the training progress were shown to the participant and briefly discussed, along with addressing any potential questions.

### 2.4 Data processing

#### 2.4.1 Psychological variables

The analysis was carried out considering the within-subject variations. Test score averages calculated before and after each condition (CTRL and BF) and were also used as indexes of biofeedback-training induced changes.

#### 2.4.2 Physiological variables

Data analysis was carried out considering intra-individual variations of physiological parameters between (i.e., metabolism level) and within the same evaluation session (i.e., hydration), in the stress and recovery phases (Time). In order to ensure a standardised comparison among participants, we employed min-max normalisation for all physiological signals.^2^ Subsequently, we determined the baseline value, which served as a reference for the subject’s resting physiological state, recorded, as mentioned, during a 4-min period at the beginning of each session (Damasio, 1998). This baseline was established by calculating the mean values of the physiological parameters during the resting period. These mean values were then subtracted from the peak values recorded for the same parameters during the stress tasks and recovery periods within the assessment framework. This procedure helped to isolate the effects of the stress and recovery activities on the physiological parameters by providing a normalised reference point against which changes can be measured. Subsequently, baseline amplitude was calculated over 2 min rest data recording to provide an indication of the ongoing rest activity (Damasio, 1998).

The main metric used for presenting the efficacy of self-regulation is the difference (i.e., *Delta*) calculated between the mean of the baseline and peak values of physiological parameters during both stress tasks and recovery periods during the assessment.

### 2.5 Statistical analysis

We faced a significant challenge in recruiting participants due to the specific nature of our target population. The strict inclusion criteria, which were essential to ensure that participants represented the level of performance relevant to our research objectives, inevitably limited the pool of available subjects. Consequently, this led to an unavoidable limitation of our study: the inability to recruit a larger cohort, resulting in a small sample size. Thus, it was necessary to assess the statistical power of our study, given the actual number of participants and the observed effect sizes. To this end, a post-hoc power analysis was performed using G*Power software. The results suggested that, to achieve a power of 0.80 (i.e., the minimum acceptable level), which indicates a Type II error rate (β) of 0.20, we would have needed a sample size of approximately 12 participants. This calculation of β was reported for each statistical test conducted in our study, including the repeated measures ANOVAs and paired *t*-tests, to provide a comprehensive understanding of the power dynamics at play in our research.

The delta-normalised physiological parameters were meticulously analysed to gain insights into the differential impacts of the experimental conditions. Thus, we conducted five separate 2 (Condition: Control vs. BF) × 2 (Time: stress vs. recovery) repeated measures Analysis of Variance (ANOVA), with the primary aim of discerning both the individual and interactive effects of Condition and Time on each of the dependent variables. This method has allowed the examination of both main effects and the interaction between *time* and *condition* for each dependent variable. This was followed by post-hoc tests specifically designed to compare means between treatments (CTRL and BF) for each phase individually, providing a detailed view of treatment effects in different contexts. Bonferroni correction was applied to probability values to account for potential biases due to multiple comparisons.

The psychological measures (i.e., the scores on the MAIA questionnaire) were initially analysed using a series of paired sample *t*-tests to assess whether any noteworthy improvements occurred between the baseline and control phases. However, the results showed no significant improvements. Subsequently, the focus of our analysis shifted to explicitly investigating the effects of treatment. This was achieved by conducting a comparative analysis between the two conditions, CTRL and BF. This comparative approach was crucial in isolating and understanding the specific effects of the BF treatment. Prior to any statistical tests, descriptive statistics and assumptions were calculated for all data and are provided in the Supplementary information. Note that the Shapiro–Wilk test indicated that all measures had a normal distribution (*p* > 0.05).

## 3 Results

### 3.1 Psychological variables

**Figure 2:**
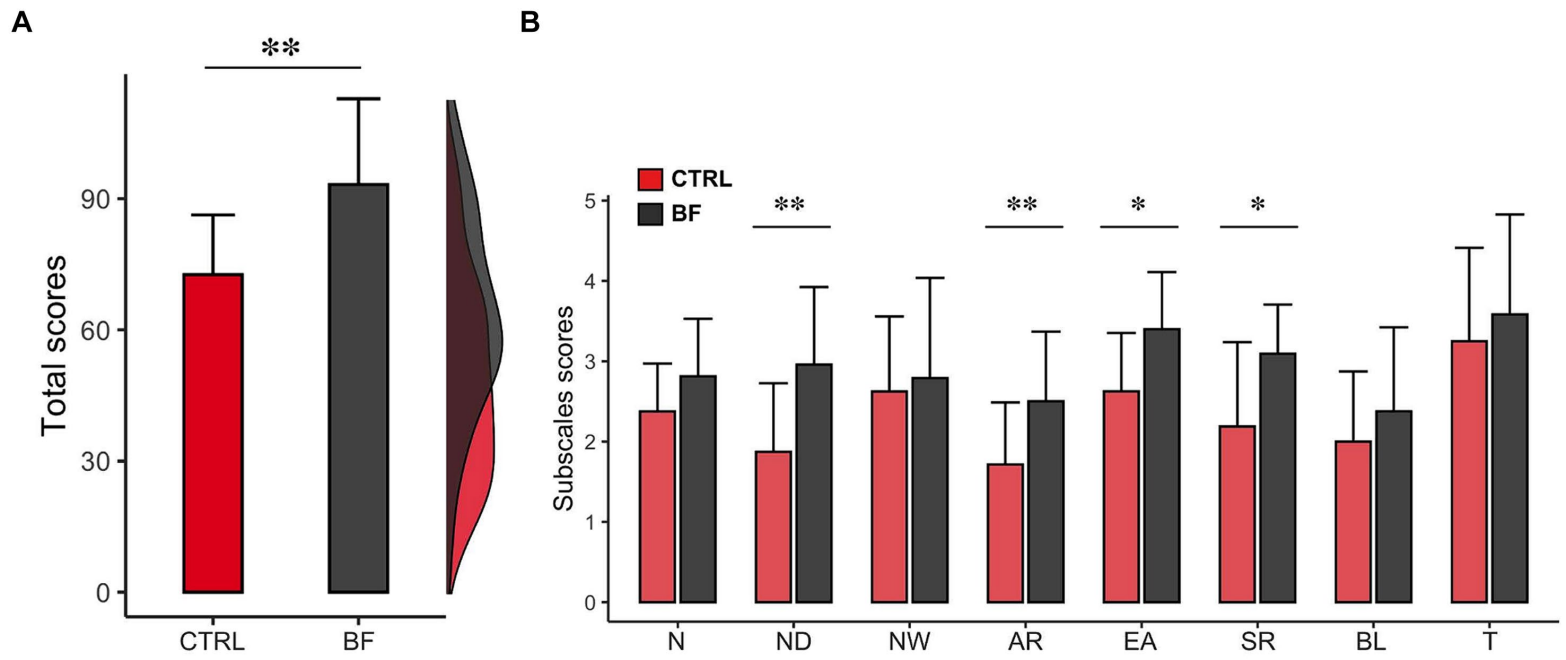
MAIA questionary data. Image shows results after BF treatment (black) and control (red) conditions. The results are presented for **(A)** total score and **(B)** questionnaire’s subscales. N, Noticing; ND, Not Distracting; NW, Not Worrying; AR, Attention Regulation; EA, Emotional Awareness; SR, Self-regulation; BL, Body Listening; T, Trusting. ** *p* < 0.001; * *p* < 0.05 Significant differences.

As shown in Table 2, analysis revealed significant differences between conditions (CTRL vs. BF) on *Total scores* (*t_(7)_* = −4.26, *p* < 0.001, *d =* −1.50, *1−β* = 0.82), as well as on *Not Distracting* (*t_(7)_* = −4.66, *p* < 0.001, *d* = −1.65, *1−β* = 0.82), *Attention regulation* (*t_(7)_* = −3.90, *p* < 0.05, *d* = −1.38, *1−β* = 0.64), *Emotional awareness* (*t_(7)_* = −2.78, *p* < 0.05, *d* = −1.98, *1−β* = 0.74) and *Self-regulation* subscales (*t_(7)_* = −3.07, *p* < 0.05, *d* = −1.09, *1−β* = 0.67) (see Figure 2).

**Table 2:** Results of a paired simple *t*-test on psychological parameters.

	*t*	df	*p*	Cohen’s d	1−β
Total score	−4.26	7	< 0.001	−1.50	0.82
Noticing	−1.42	7	0.25	−0.50	0.36
Not distracting	−4.66	7	< 0.001	−1.65	0.82
Not worrying	−0.42	7	0.69	−0.15	0.065
Attention regulation	−3.90	7	< 0.01	−1.38	0.64
Emotional awareness	−2.78	7	< 0.05	−0.98	0.74
Self-regulation	−3.07	7	< 0.05	−1.09	0.67
Body listening	−0.86	7	0.42	−0.30	0.16
Trusting	−1.47	7	0.18	−0.52	0.10

### 3.2 Physiological variables

**Figure 3:**
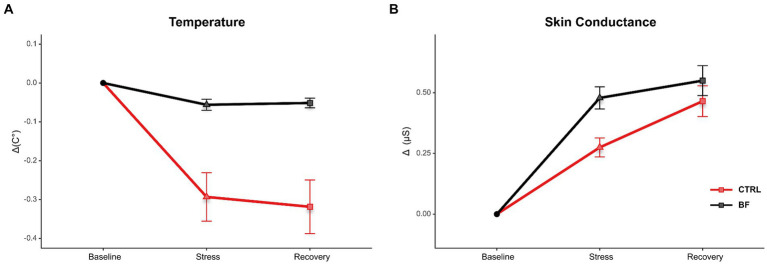
Effects of treatment. Images show the effects of treatment in peripheral temperature **(A)** and skin conductance level **(B)** in BF (black) and CTRL (red) conditions during baseline, stress, and recovery phase. Vertical bars represent standard errors.

Repeated measures ANOVA 2 (Conditions: CTRL vs. BF) × 2 (Time: stress and recovery; within-subjects) was performed to assess the effect of treatment on their self-regulatory ability (Figure 3).

Repeated measures ANOVA showed significant effects of condition (CTRL vs. BF) for the parameters *T* (*F_(1,7)_* = 17.45, *p* < 0.001, *η^2^_p_* = 0.71, *1−β* = 1.00) and for *BVP* (*F_(1,7)_* = 15.05, *p* < 0.05, *η^2^_p_* = 0.68, *1−β* = 1.00). While the difference for the *SCL* has not reached statistical significance, the direction and the amplitude of change indicate positive effects of BF (*F_(1,7)_* = 2.88, *p* > 0.05, *η^2^_p_* = 0.29), as it is in the case of *EMG* (*F*_(1,7)_ = 1.88, *p* > 0.05, *η^2^_p_* = 0.21). Additionally, significant effects for Time were recognised in parameter *SCL* (*F_(1,7)_* = 20.40, *p* < 0.001, *η^2^_p_* = 0.74, *1−β* = 1.00). We observed significant interaction between Condition and Time for parameter *T* (*F_(1,7)_* = 10.16, *p* < 0.05, *η^2^_p_* = 0.59, *1−β* = 1.00). Post-hoc analysis revealed that the *T* was significantly higher after the treatment (BF) in both stress (*t_(7)_* = 3.92, *d* = −1.77, *p* < 0.05) and recovery periods (*t*_(7)_ = −4.41, *d* = −1.99, *p* < 0.05). We did not observe any significant differences for other parameters (Table 3).

**Table 3:** Results of the repeated measures ANOVAs on physiological parameters.

Parameter	Cases	Sum of squares	df	Mean square	*F*	*p*	η^2^_p_	1−β
T	Condition × Time	0.00	7	0.00	10.16	< 0.05	0.59	1.00
	Condition	0.51	7	0.51	17.45	< 0.001	0.71	1.00
	Time	8.601 × 10^–4^	7	8.601 × 10^–4^	3.86	0.09	0.36	0.95
SC	Condition × Time	0.03	7	0.03	3.54	0.10	0.34	0.933
	Condition	0.17	7	0.17	2.88	0.13	0.29	0.87
	Time	0.14	7	0.14	20.40	< 0.001	0.74	1.00
EMG	Condition × Time	0.04	7	0.04	1.32	0.29	0.16	0.56
	Condition	0.34	7	0.34	1.88	0.21	0.21	0.71
	Time	0.01	7	0.01	0.16	0.70	0.02	0.11
BVP	Condition × Time	0.00	7	0.00	0.39	0.55	0.05	0.20
	Condition	0.05	7	0.05	15.05	< 0.01	0.68	1.00
	Time	0.01	7	0.01	2.06	0.19	0.23	0.75
RR	Condition × Time	7.494 × 10^–4^	7	7.494 × 10^–4^	0.03	0.86	0.01	0.08
	Condition	0.04	7	0.04	0.51	0.50	0.07	0.27
	Time	0.11	7	0.11	4.51	0.07	0.39	0.97

Abbreviations for the metrics are as follows: T, peripheral temperature; SCL, skin conductance level; EMG, muscle activity; BVP, blood volume pressure and heart rate; RR, respiration rate.

## 4 Discussion

In the present study, we investigated for the first time whether training self-regulatory skills through BF can influence resilience to stressful conditions in prepubescent elite female athletes. Our results show that BF training in elite athletes (1) increased self-regulation and psychological resilience under stressful conditions and (2) it seemingly decreased sensitivity to gymnastics-specific stress. Taken together, these two findings support the idea that psychophysiological self-regulation is trainable.

The protocol of this study was based on the previous research that demonstrated the efficacy of thermal and electrodermal (Peper and Schmid, 1983; Rusciano et al., 2017; Iodice et al., 2022) biofeedback in the case of self-regulation training for the gymnasts. Our results confirm the positive influence of BF training on both psychological and physiological dimensions of self-regulation. In particular, the former was assessed using the MAIA test, which has been used extensively in studies of both healthy and clinical populations (Park et al., 2021; Phillipou et al., 2022) and to assess training-induced changes in the case of mind–body interventions such as meditation (Bornemann et al., 2015) and biofeedback (Iodice et al., 2022). Indeed, while all the factors showed improvements after the BF training, statistical significance was found for more sophisticated interoceptive awareness sub-abilities such as not-distraction (the tendency not to use distraction to cope with discomfort), attention regulation (the ability to maintain and control attention to bodily sensations), emotion awareness (the ability to attribute specific bodily sensations to physiological manifestations of emotions), and self-regulation (the ability to regulate distress through attention to bodily sensations). On the contrary, sub-abilities that can be considered as precursors of these more sophisticated ones (such as noticing or the ability to be aware of body sensations, or body listening, i.e., the tendency to actively listen to the body for insights) were not significantly increased after training. This is partly in contrast to Bornermann and colleagues who reported a significant increase in all the sub-abilities after three months of contemplative training (Bornemann et al., 2015). A possible explanation for this difference may lie in the intrinsic differences between the two employed training procedures. The authors of the study required participants to complete structured modules based on meditation techniques during which no objective feedback were provided (thus stimulating participants’ subjective ability to predict their physiological state), our training was based on objectification of internal physiological state (thus avoiding participants’ to firstly focus on “recognising” phase (e.g., noticing and body listening), concentrating their resources on training their ability to modify physiological signals, thus skipping faster to the “regulating phase”) (Bornemann et al., 2015).

Similarly, Lima-Araujo et al. (2022) reported a significant increase in five of the eight MAIA sub-abilities (i.e., Body Listening, Trusting, Self-Regulation, Attention Regulation and Emotional Awareness) following a brief mindfulness training that focused heavily on body scan abilities and learning of breathing patterns. Although the participants in their training did not receive objective feedback on their performance, this protocol nonetheless presents many similarities to the one we used, which may explain the greater overlap between our respective results.

Also, it should be considered that the uniquely female composition of our sample did not allow us to test and/or control for possible effects related to gender difference. Recent evidence has in fact shown that gender can influence each of the sub-components of interoception and therefore a comparison between the responses in the two genders could have possibly helped to explain why lower-level skills (e.g., noticing; not worrying) did not significantly differ after training. For example, Grabauskaitė et al. (2017) showed significant differences on some sub-components in female and male participants. In particular, male participants reported higher values on the not worrying and trusting dimensions, whereas female participants reported higher scores on emotional awareness and body listening. Additionally, children’s ability to handle internal body cues based on interoceptive awareness typically improves as they grow and develop (Schmitt and Schoen, 2022). In particular, children are expected to present five stages of maturing of their interoceptive awareness capacity: (i) Noticing—the child notices that the interoceptive sensation is occurring; (ii) Naming—the child is able to label and describe the interoceptive sensation; (iii) Linking—the child is able to connect an emotions to the interoceptive sensation; (iv) Understanding and recognising the impact—the child understands the impact of the interoceptive sensation and this requires the second-order cognitive skills of reasoning, reflection, organization and planning; (v) Handling, managing and coping—the child needs to be able to cope with the interoceptive sensation and regulate it (Cheung et al., 2023). Therefore, another possibility to consider is that given the age of our sample (i.e., pre-pubescent), the participants did not show post-training differences with respect to the low-level components, as these components were already acquired and mastered.

Furthermore, our results may contribute to the ongoing debate about the components of interoception—accuracy, sensitivity and awareness—and their relationships, as recently highlighted by Garfinkel et al. (2015). Indeed, the authors suggest that the aforementioned categorization could be reconceptualised in terms of a distinction between *objective* interoceptive processes (i.e., accuracy: the ability to correctly monitor changes in internal body state), *subjective* (i.e., sensibility: the tendency to focus on internal body state), and *metacognitive* process (i.e., awareness or error awareness: quantifiable difference between self-reported judgement of interoceptive accuracy and objectively assessed interoceptive accuracy). This idea is also seemingly supported by recent findings suggesting differences in the neural underpinnings of different interoceptive components (Du et al., 2023), feedback-based training bases its effectiveness (at least in part) on its potential to highlight errors in the interoceptive monitoring process, allowing the subject to immediately compare the actual (real) state with the perceived one, thus allowing for continuous adjustment of the predicted state. This focus on the *metacognitive* component of interoception is the distinctive feature that distinguishes neuro- and biofeedback procedures from other methods commonly used to train the ability to consciously manage psychophysiological states. As a result, our findings contribute to the under-documented literature on interoceptive processes and feedback-based training in the young athletic population.

Indeed, while the efficacy of the BF-based procedures has been widely studied in clinical contexts (e.g., ADHD, Kuznetsova et al., 2022), and their usefulness was recently confirmed in a systematic review of the effects of biofeedback training in children and adolescents (Dormal et al., 2021), their potential application in the case of young athletes is still relatively unexplored (see Zadkhosh et al., 2018). The recorded physiological parameters show a main effect of condition (CTRL vs. BF, *p* < 0.001) for the temperature parameter. In particular, our data suggest a lower sensitivity to the stress stimuli after BF training. We also note that in the control condition, before training with BF, prepubescent female athletes were not able to regain the homeostatic balance, once perturbed by the stress stimuli.

On the other hand, while the overall trend manifests the expected behaviour, the observed changes in the skin conductance level parameter did not reach statistical difference, nor did the interaction between the experimental conditions. Our best *a posteriori* hypothesis regarding such data concerns the nature of the physiological signals investigated and the age of the participants. Indeed, while the concept of body temperature is relatively straightforward to grasp., conductance is a more abstract notion. Referring to classical theories of child development (Piaget, 1952), it would appear that, from this point of view, the participants in this study are at the end of the concrete operations stage (or at the very beginning of the formal stage). At this stage, the ability to use abstract concepts is not yet fully acquired.

Therefore, our findings firstly corroborate those of previous studies suggesting that when the subjects receive interoceptive feedback on their neurovisceral state during targeted training, it allows for the adaptation of self-regulatory control mechanisms (e.g., Mirifar et al., 2017; Meyerholz et al., 2019). Furthermore, our study extends the findings on the influence of self-regulatory capacity on the stress management process to prepubertal athletes and contributes to the understanding of the relationship between stress, psychophysiological responses and training capacity. Finally, we suggest that our approach introduces a new procedure for training young elite athletes to protect their psychophysical balance during the long training sessions (24 h per week in our sample), which could be crucial for injury prevention, as suggested by some authors (Rusciano et al., 2017). Our study demonstrates the effectiveness of biofeedback training in improving elite athletes’ self-regulation and psychological resilience, particularly their ability to cope with stress-related challenges. This training appears to reduce their sensitivity to gymnastics-specific stressors, highlighting its potential benefits in a sporting context.

Furthermore, our study emphasizes the importance of psychophysiological self-regulation, which involves an individual’s ability to manage emotional and cognitive states while adapting to different environmental conditions. This ability is crucial for maintaining balance in response to complex situational demands. In summary, our research supports the idea that interoceptive feedback training can significantly enhance an individual’s self-regulatory mechanisms.

## Data availability statement

The raw data supporting the conclusions of this article will be made available by the authors, without undue reservation.

## Ethics statement

The studies involving humans were approved by the Ethical Committee of the University of Rouen Normandy, France, Protocol n. 2020-04-A. The studies were conducted in accordance with the local legislation and institutional requirements. Written informed consent for participation in this study was provided by the participants’ legal guardians/next of kin.

## Author contributions

GP: Conceptualization, Investigation, Writing – original draft, Methodology. MB: Data curation, Supervision, Writing – review & editing. AC: Data curation, Formal analysis, Software, Writing – review & editing. LC: Writing – original draft, Data curation, Formal analysis, Methodology. RP: Supervision, Validation, Visualization, Writing – review & editing. RT: Conceptualization, Methodology, Supervision, Validation, Writing – review & editing. PI: Conceptualization, Investigation, Methodology, Project administration, Resources, Supervision, Validation, Writing – original draft, Writing – review & editing.

## Footnotes

^1^The recruitment was performed in January 2020. The data collection started in February and lasted until June of the same year.

^2^This approach is essential for adjusting for inter-individual variances in physiological data (Morgan, 1974; Arnold and Fletcher, 2021). The normalisation was performed for each data point and each parameter, using the following equation:

$$Xn=\left(X-X_{min}\right)/\left(X_{max}-X_{min}\right)$$

Here *Xn* denotes the value of the signal following min-max normalisation. *X* represents the original, or raw, signal value, *Xmax* is the maximum value observed in the signal, and *Xmin* indicates the minimum value within the signal.

## Glossary

CTRL: Control condition
BF: Biofeedback condition
T: Peripheral temperature
SCL: Skin Conductance Level
EMG: Muscle activity
BVP: Blood Volume Pressure
RR: Respiration Rate
W: Weekly
MAIA: Multidimensional Assessment of Interoceptive Awareness
N: Noticing
ND: Not Distracting
NW: Not Worrying
AR: Attention Regulation
EA: Emotional Awareness
SR: Self-regulation
BL: Body Listening
T: Trusting

## References

[ref1] Anderson G. S. Di Nota P. M. Metz G. A. S. Andersen J. P. (2019). The impact of acute stress physiology on skilled motor performance: implications for policing. Front. Psychol. 10:10. doi: 10.3389/fpsyg.2019.02501, PMID: 31781001 PMC6856650

[ref2] Arena J. G. Bruno G. M. Hannah S. L. Meador K. J. (1995). A comparison of frontal electromyographic biofeedback training, trapezius electromyographic biofeedback training, and progressive muscle relaxation therapy in the treatment of tension headache. Headache 35, 411–419. doi: 10.1111/j.1526-4610.1995.hed3507411.x, PMID: 7672959

[ref3] Arnold R. Fletcher D., editors. Stress, well-being, and performance in sport. New York: Routledge; (2021)

[ref4] Balk Y. A. Englert C. (2020). Recovery self-regulation in sport: theory, research, and practice. Int. J. Sports Sci. Coach. 15, 273–281. doi: 10.1177/1747954119897528

[ref5] Bar-Eli M. Dreshman R. Blumenstein B. Weinstein Y. (2002). The effect of mental training with biofeedback on the performance of young swimmers. Appl. Psychol. 51, 567–581. doi: 10.1111/1464-0597.00108

[ref6] Benarroch E. E. Benjamini Y. Hochberg Y. (1995). Controlling the false discovery rate: a practical and powerful approach to multiple testing. Mayo Clin. Proc. 57, 289–300.

[ref7] Blumenstein B. Breslav I. Bar-Eli M. Tenenbaum G. Weinstein Y. (1995). Regulation of mental states and biofeedback techniques: effects on breathing pattern. Biofeedback Self-Regul. 20, 169–183. doi: 10.1007/BF01720972, PMID: 7662752

[ref8] Blumenstein B. Hung T. M. (2014). “Self-regulation and biofeedback” in Routledge companion to sport and exercise psychology, global perspectives and fundamental concepts. eds. Papaioannou A. Hackfort D. (London: Routledge)

[ref9] Blumenstein B. Weinstein Y. (2011). Biofeedback training: enhancing athletic performance. Biofeedback 39, 101–104. doi: 10.5298/1081-5937-39.3.07

[ref10] Bornemann B. Herbert B. M. Mehling W. E. Singer T. (2015). Differential changes in self-reported aspects of interoceptive awareness through 3 months of contemplative training. Front. Psychol. 5:5. doi: 10.3389/fpsyg.2014.01504 PMC4284997 25610410

[ref11] Boucsein W. Electrodermal activity. New York, NY: Springer; (2012)

[ref12] Brown B. B. (1977). Stress and the art of biofeedback. Oxford: Harper & Row.

[ref13] Caine D. J. Nassar L. (2005). “Gymnastics injuries” in Epidemiology of pediatric sports injuries. eds. Caine D. J. Maffulli N. (Basel: Karger Publishers) 48, 18–58. 10.1159/000084282 16247252

[ref14] Cañal-Bruland R. Pijpers J. R. Oudejans R. R. (2010). The influence of anxiety on action-specific perception. Anxiety Stress Coping 23, 353–361. doi: 10.1080/10615800903447588, PMID: 19946816

[ref15] Candia-Rivera D. Catrambone V. Thayer J. F. Gentili C. Valenza G. (2022). Cardiac sympathetic-vagal activity initiates a functional brain–body response to emotional arousal. Proc. Natl. Acad. Sci. 119:e2119599119. doi: 10.1073/pnas.2119599119, PMID: 35588453 PMC9173754

[ref16] Cheung H. Y. L. Brown T. Yu M. L. Cheung P. P. (2023). The relationship between school-age Children’s self-reported perceptions of their interoceptive awareness and emotional regulation: an exploratory study. J. Occup. Ther. Sch. Early Interv. doi: 10.1080/19411243.2023.2215764

[ref17] Codonhato R. Rubio V. Oliveira P. M. P. Resende C. F. Rosa B. A. M. Pujals C.. (2018). Resilience, stress and injuries in the context of the Brazilian elite rhythmic gymnastics. PLoS One 13:e0210174. doi: 10.1371/journal.pone.0210174, PMID: 30596793 PMC6312305

[ref18] Damasio A. R. (1998). Emotion in the perspective of an integrated nervous system. Brain Res. Rev. 26, 83–86. doi: 10.1016/S0165-0173(97)00064-7, PMID: 9651488

[ref19] Davis P. A. Sime W. E. (2005). Toward a psychophysiology of performance: sport psychology principles dealing with anxiety. Int. J. Stress. Manag. 12, 363–378. doi: 10.1037/1072-5245.12.4.363

[ref20] Dormal V. Vermeulen N. Mejias S. (2021). Is heart rate variability biofeedback useful in children and adolescents? A systematic review. J. Child Psychol. Psychiatry 62, 1379–1390. doi: 10.1111/jcpp.13463, PMID: 34155631

[ref21] Du X. Li Q. Xiang G. Xiao M. Liu X. Chen X.. (2023). The relationship between brain neural correlates, self-objectification, and interoceptive sensibility. Behav. Brain Res. 439:114227. doi: 10.1016/j.bbr.2022.114227, PMID: 36436730

[ref22] Duda J. Gano-Overway L. A. (1996a). Anxiety in elite young gymnasts: part I definitions of stress and relaxation. USA Gymnast. Online Tech. 16, 22–24.

[ref23] Duda J. Gano-Overway L. A. (1996b). Anxiety in elite young gymnasts: part II–sources of stress. USA Gymnast. Online Tech. 16, 4–6.

[ref24] Dugdale J. R. Eklund R. C. Gordon S. (2002). Expected and unexpected stressors in major international competition: appraisal, coping, and performance. Sport Psychol. 16, 20–33. doi: 10.1123/tsp.16.1.20

[ref25] Dupee M. Forneris T. Werthner P. (2016). Perceived outcomes of a biofeedback and neurofeedback training intervention for optimal performance: learning to enhance self-awareness and self-regulation with Olympic athletes. Sport Psychol. 30, 339–349. doi: 10.1123/tsp.2016-0028

[ref26] Dupee M. Werthner P. (2011). Managing the stress response: the use of biofeedback and neurofeedback with Olympic athletes. Biofeedback 39, 92–94. doi: 10.5298/1081-5937-39.3.02

[ref27] Dworkin B. R. Elbert T. Rau H. Birbaumer N. Pauli P. Droste C.. (1994). Central effects of baroreceptor activation in humans: attenuation of skeletal reflexes and pain perception. Proc. Natl. Acad. Sci. USA 91, 6329–6333. doi: 10.1073/pnas.91.14.6329, PMID: 8022781 PMC44195

[ref28] Ely F. Munroe-Chandler K. Jenny O. McCullagh P. (2020). The practice of imagery: a review of 25 years of applied sport imagery recommendations. J. Imag. Res. Sport Phys. Act. 15:20200018. doi: 10.1515/jirspa-2020-0018

[ref29] Ferguson K. N. Hall C. Divine A. (2020). Examining the effects of an interspersed biofeedback training intervention on physiological indices. Sport Psychol. 34, 310–318. doi: 10.1123/tsp.2019-0111

[ref30] Fletcher D. Hanton S. (2003). Sources of organizational stress in elite sports performers. Sport Psychol. 17, 175–195. doi: 10.1123/tsp.17.2.175

[ref31] Galloway S. M. (2011). The effect of biofeedback on tennis service accuracy. Int. J. Sport Exerc. Psychol. 9, 251–266. doi: 10.1080/1612197X.2011.614851

[ref32] Garfinkel S. N. Seth A. K. Barrett A. B. Suzuki K. Critchley H. D. (2015). Knowing your own heart: distinguishing interoceptive accuracy from interoceptive awareness. Biol. Psychol. 104, 65–74. doi: 10.1016/j.biopsycho.2014.11.004, PMID: 25451381

[ref33] Gillen Z. M. Housh T. J. Schmidt R. J. Herda T. J. De Ayala R. J. Shoemaker M. E.. (2021). Comparisons of muscle strength, size, and voluntary activation in pre- and post-pubescent males and females. Eur. J. Appl. Physiol. 121, 2487–2497. doi: 10.1007/s00421-021-04717-1, PMID: 34032904

[ref34] Grabauskaitė A. Baranauskas M. Griškova-Bulanova I. (2017). Interoception and gender: what aspects should we pay attention to? Conscious. Cogn. 48, 129–137. doi: 10.1016/j.concog.2016.11.002, PMID: 27866005

[ref35] Granados A. Gebremariam A. Lee J. M. (2015). Relationship between timing of peak height velocity and pubertal staging in boys and girls. J. Clin. Res. Pediatr. Endocrinol. 7, 235–237. doi: 10.4274/jcrpe.2007, PMID: 26831559 PMC4677560

[ref36] Greenspan M. J. Feltz D. L. (1989). Psychological interventions with athletes in competitive situations: a review. Sport Psychol. 3, 219–236. doi: 10.1123/tsp.3.3.219

[ref37] Howard S. J. Williams K. E. (2018). Early self-regulation, early self-regulatory change, and their longitudinal relations to adolescents’ academic, health, and mental well-being outcomes. J. Dev. Behav. Pediatr. 39, 489–496. doi: 10.1097/DBP.0000000000000578, PMID: 29781830

[ref38] Inzlicht M. Werner K. M. Briskin J. L. Roberts B. W. (2021). Integrating models of self-regulation. Annu. Rev. Psychol. 72, 319–345. doi: 10.1146/annurev-psych-061020-105721 33017559

[ref39] Iodice P. Cannito L. Chaigneau A. Palumbo R. (2022). Learned self-regulation in top-level managers through neurobiofeedback training improves decision making under stress. Sci. Rep. 12:6127. doi: 10.1038/s41598-022-10142-x, PMID: 35414098 PMC9005532

[ref40] Iodice P. Porciello G. Bufalari I. Barca L. Pezzulo G. (2019). An interoceptive illusion of effort induced by false heart-rate feedback. Proc. Natl. Acad. Sci. USA 116, 13897–13902. doi: 10.1073/pnas.1821032116, PMID: 31235576 PMC6628799

[ref41] Jones M. V. (2003). Controlling emotions in sport. Sport Psychol. 17, 471–486. doi: 10.1123/tsp.17.4.471

[ref42] Jurimae T. Jurimae J. Growth, physical activity, and motor development in prepubertal children. Boca Raton, FL: CRC Press; (2001)

[ref43] Kennedy L. Parker S. H. (2019). Biofeedback as a stress management tool: a systematic review. Cognit. Technol. Work 21, 161–190. doi: 10.1007/s10111-018-0487-x

[ref44] Khazan I. Z. The clinical handbook of biofeedback: a step-by-step guide for training and practice with mindfulness. Hoboken, NJ: Wiley-Blackwell; (2013)

[ref45] Kuznetsova E. Veilahti A. V. P. Akhundzadeh R. Radev S. Konicar L. Cowley B. U. (2022). Evaluation of neurofeedback learning in patients with ADHD: a systematic review. Appl. Psychophysiol. Biofeedback 48, 11–25. doi: 10.1007/s10484-022-09562-2 36178643 PMC9908642

[ref46] Laborde S. Mosley E. Thayer J. F. (2017). Heart rate variability and cardiac vagal tone in psychophysiological research-recommendations for experiment planning, data analysis, and data reporting. Front. Psychol. 8:213. doi: 10.3389/fpsyg.2017.00213 28265249 PMC5316555

[ref47] Landers D. M. (1985). “Psychophysiological assessment and biofeedback” in Biofeedback and sports science. eds. Sandweiss J. H. Wolf S. L. (Boston, MA: Springer)

[ref48] Lehrer P. M. Vaschillo E. Vaschillo B. (2000). Resonant frequency biofeedback training to increase cardiac variability: rationale and manual for training. Appl. Psychophysiol. Biofeedback 25, 177–191. doi: 10.1023/A:1009554825745 10999236

[ref49] Lehrer P. Vaschillo B. Zucker T. Graves J. Katsamanis M. Aviles M.. (2013). Protocol for heart rate variability biofeedback training. Biofeedback 41, 98–109. doi: 10.5298/1081-5937-41.3.08

[ref50] Lima-Araujo G. L. de Sousa Júnior G. M. Mendes T. Demarzo M. Farb N. Barros de Araujo D.. (2022). The impact of a brief mindfulness training on interoception: a randomized controlled trial. PLoS One 17:e0273864. doi: 10.1371/journal.pone.0273864, PMID: 36070308 PMC9451078

[ref51] Lochbaum M. Stoner E. Hefner T. Cooper S. Lane A. M.. (2022). Sport psychology and performance meta-analyses: a systematic review of the literature. PLoS One 17:e0263408. doi: 10.1371/journal.pone.0263408, PMID: 35171944 PMC8849618

[ref52] Marin M. F. Lord C. Andrews J. Juster R. P. Sindi S. Arsenault-Lapierre G.. (2011). Chronic stress, cognitive functioning and mental health. Neurobiol. Learn. Mem. 96, 583–595. doi: 10.1016/j.nlm.2011.02.016 21376129

[ref53] Mariotti A. (2015). The effects of chronic stress on health: new insights into the molecular mechanisms of brain-body communication. Future Sci. 1, 2–4. doi: 10.4155/fso.15.21 PMC5137920 28031896

[ref54] McHugh B. Dawson N. Scrafton A. Asen E. (2010). ‘Hearts on their sleeves’: the use of systemic biofeedback in school settings. J. Fam. Ther. 32, 58–72. doi: 10.1111/j.1467-6427.2009.00486.x

[ref55] Mehling W. E. Price C. Daubenmier J. J. Acree M. Bartmess E. Stewart A. (2012). The multidimensional assessment of interoceptive awareness (MAIA). PLoS One 7:e48230. doi: 10.1371/journal.pone.0048230, PMID: 23133619 PMC3486814

[ref56] Meyerholz L. Irzinger J. Witthöft M. Gerlach A. L. Pohl A. (2019). Contingent biofeedback outperforms other methods to enhance the accuracy of cardiac interoception: a comparison of short interventions. J. Behav. Ther. Exp. Psychiatry 63, 12–20. doi: 10.1016/j.jbtep.2018.12.002, PMID: 30557753

[ref57] Mirifar A. Beckmann J. Ehrlenspiel F. (2017). Neurofeedback as supplementary training for optimizing athletes’ performance: a systematic review with implications for future research. Neurosci. Biobehav. Rev. 75, 419–432. doi: 10.1016/j.neubiorev.2017.02.005, PMID: 28185873

[ref58] Moffitt T. E. Arseneault L. Belsky D. Dickson N. Hancox R. J. Harrington H.. (2011). A gradient of childhood self-control predicts health, wealth, and public safety. Proc. Natl. Acad. Sci. 108, 2693–2698. doi: 10.1073/pnas.1010076108, PMID: 21262822 PMC3041102

[ref59] Morgan W. P. (1974). Selected psychological considerations in sport. Res. Q. 45, 374–390. doi: 10.1080/10671315.1974.10615285, PMID: 4531653

[ref60] Nippert A. H. Smith A. M. (2008). Psychologic stress related to injury and impact on sport performance. Phys. Med. Rehabil. Clin. N. Am. 19, 399–418. doi: 10.1016/j.pmr.2007.12.003, PMID: 18395654

[ref61] O’Connor D. B. Thayer J. F. Vedhara K. (2021). Stress and health: a review of psychobiological processes. Annu. Rev. Psychol. 72, 663–688. doi: 10.1146/annurev-psych-062520-122331 32886587

[ref62] Oudejans R. R. D. Kuijpers W. Kooijman C. C. Bakker F. C. (2011). Thoughts and attention of athletes under pressure: skill-focus or performance worries? Anxiety Stress Coping 24, 59–73. doi: 10.1080/10615806.2010.481331 20425657

[ref63] Park Y. L. Hunter J. Sheldon B. L. Sabourin S. DiMarzio M. Khazen O.. (2021). Pain and interoceptive awareness outcomes of chronic pain patients with spinal cord stimulation. Neuromodulation 24, 1357–1362. doi: 10.1111/ner.13318 33191569

[ref64] Paul M. Garg K. Singh Sandhu J. (2012). Role of biofeedback in optimizing psychomotor performance in sports. Asian J. Sports Med. 3 Available at: https://brieflands.com/articles/asjsm-76716.html 10.5812/asjsm.34722 PMC3307964 22461963

[ref65] Peek C. J. (2017). “A primer of traditional biofeedback instrumentation” in Biofeedback: a practitioner’s guide. eds. Schwartz M. S. Andrasik F. (New York, NY: Guilford Publications)

[ref66] Peper E. Harvey R. Tylova H. (2006). Stress protocol for assessing computer-related disorders. Biofeedback 34:58.

[ref67] Peper E. Schmid P. (1983). The use of electrodermal biofeedback for peak performance training. Somatics IV, 16–18.

[ref68] Peper E. Shaffer F. (2010). Biofeedback history: an alternative view. Biofeedback 38, 142–147. doi: 10.5298/1081-5937-38.4.03

[ref69] Perry F. D. Shaw L. Zaichkowsky L. (2011). Biofeedback and neurofeedback in sports. Biofeedback 39, 95–100. doi: 10.5298/1081-5937-39.3.10

[ref70] Pezzulo G. Iodice P. Barca L. Chausse P. Monceau S. Mermillod M. (2018). Increased heart rate after exercise facilitates the processing of fearful but not disgusted faces. Sci. Rep. 8:398. doi: 10.1038/s41598-017-18761-5, PMID: 29321533 PMC5762722

[ref71] Phillipou A. Rossell S. L. Castle D. J. Gurvich C. (2022). Interoceptive awareness in anorexia nervosa. J. Psychiatr. Res. 148, 84–87. doi: 10.1016/j.jpsychires.2022.01.051 35121272

[ref72] Piaget J. The origins of intelligence in children. New York: International Univ. Press; (1952)

[ref73] Porges S. W. (2007). The polyvagal perspective. Biol. Psychol. 74, 116–143. doi: 10.1016/j.biopsycho.2006.06.009, PMID: 17049418 PMC1868418

[ref74] Prinzel L. J. III Pope A. T. Freeman F. G. Application of physiological self-regulation and adaptive task allocation techniques for controlling operator hazardous states of awareness. NASA Langley Res Cent Hampton VA (2001), pp. 1–22.

[ref75] Quintana D. S. Heathers J. A. (2014). Considerations in the assessment of heart rate variability in biobehavioral research. Front. Psychol. 5:805. doi: 10.3389/fpsyg.2014.00805 25101047 PMC4106423

[ref76] Robson D. A. Allen M. S. Howard S. J. (2020). Self-regulation in childhood as a predictor of future outcomes: a meta-analytic review. Psychol. Bull. 146, 324–354. doi: 10.1037/bul0000227, PMID: 31904248

[ref77] Rumbold J. Fletcher D. Daniels K. (2012). A systematic review of stress management interventions with sport performers. Sport Exerc. Perform. Psychol. 1, 173–193. doi: 10.1037/a0026628

[ref78] Rusciano A. Corradini G. Stoianov I. (2017). Neuroplus biofeedback improves attention, resilience, and injury prevention in elite soccer players. Psychophysiology 54, 916–926. doi: 10.1111/psyp.12847, PMID: 28220500

[ref79] Russo S. J. Murrough J. W. Han M. H. Charney D. S. Nestler E. J. (2012). Neurobiology of resilience. Nat. Neurosci. 15, 1475–1484. doi: 10.1038/nn.3234, PMID: 23064380 PMC3580862

[ref80] Sandweiss J. H. Wolf S. L., editors. Biofeedback and sports science. Boston, MA: Springer; (1985)

[ref81] Schmitt C. M. Schoen S. (2022). Interoception: a multi-sensory foundation of participation in daily life. Front. Neurosci. 16:875200. doi: 10.3389/fnins.2022.875200, PMID: 35757546 PMC9220286

[ref82] Schwartz M. S. Andrasik F., editors. Biofeedback: a practitioner’s guide. New York, NY: Guilford Publications; (2017).

[ref83] Schwartz M. S. Collura T. F. Kamiya J. Schwartzthe N. M. (2017). “History and definitions of biofeedback and applied psychophysiology” in Biofeedback: a practitioner’s guide. eds. Schwartz M. S. Andrasik F. (New York, NY: Guilford Publications)

[ref84] Shaffer F. Combatalade D. Peper E. (2016). A guide to cleaner skin temperature recordings and more versatile use of your thermistor. Biofeedback 44, 168–176. doi: 10.5298/1081-5937-44.3.06

[ref85] Slavikova M. Sekaninova N. Bona O. L. Visnovcova Z. Tonhajzerova I. (2020). Biofeedback – a promising non pharmacological tool of stress – related disorders. Acta Med. Martin. 20, 1–8. doi: 10.2478/acm-2020-0001

[ref86] Smith K. E. Pollak S. D. (2020). Early life stress and development: potential mechanisms for adverse outcomes. J. Neurodev. Disord. 12:34. doi: 10.1186/s11689-020-09337-y, PMID: 33327939 PMC7745388

[ref87] Strack B. Linden M. Wilson V. Biofeedback & neurofeedback applications in sport psychology. Wheat Ridge, CO: AAPB Publishing; (2011).

[ref88] Tangney J. P. Baumeister R. F. Boone A. L. (2004). High self-control predicts good adjustment, less pathology, better grades, and interpersonal success. J. Pers. 72, 271–324. doi: 10.1111/j.0022-3506.2004.00263.x, PMID: 15016066

[ref89] Thayer J. F. Lane R. D. (2009). Claude Bernard and the heart-brain connection: further elaboration of a model of neurovisceral integration. Neurosci. Biobehav. Rev. 33, 81–88. doi: 10.1016/j.neubiorev.2008.08.004 18771686

[ref90] Thoits P. A. (2010). Stress and health: major findings and policy implications. J. Health Soc. Behav. 51, S41–S53. doi: 10.1177/0022146510383499, PMID: 20943582

[ref91] Vine S. J. Moore L. J. Wilson M. R. (2016). An integrative framework of stress, attention, and visuomotor performance. Front. Psychol. 7:1671. doi: 10.3389/fpsyg.2016.01671, PMID: 27847484 PMC5088191

[ref92] White R. L. Bennie A. (2015). Resilience in youth sport: a qualitative investigation of gymnastics coach and athlete perceptions. Int. J. Sports Sci. Coach. 10, 379–393. doi: 10.1260/1747-9541.10.2-3.379

[ref93] Willem C. Gandolphe M. C. Nandrino J. L. Grynberg D. (2022). French translation and validation of the multidimensional assessment of interoceptive awareness (MAIA-FR). Can. J. Behav. Sci. 54, 234–240. doi: 10.1037/cbs0000271

[ref94] Wilson S. (2006). “Optimal Performance and Health.” In Optimizing performance and health suite. Amersfoort, The Netherlands: Biofeedback Foundation of Europe. Retrieved August 5, 2011, from: http://www.bfe.org/articles/Expert%20series%20Sue%20Wilson.pdf

[ref95] Yu B. Funk M. Hu J. Wang Q. Feijs L. (2018). Biofeedback for everyday stress management: a systematic review. Front ICT. 5:23. doi: 10.3389/fict.2018.00023

[ref96] Yu A. N. C. Iodice P. Pezzulo G. Barca L. (2021). Bodily information and top-down affective priming jointly affect the processing of fearful faces. Front. Psychol. 12:625986. doi: 10.3389/fpsyg.2021.625986, PMID: 34149514 PMC8206275

[ref97] Zaccaro A. Piarulli A. Laurino M. Garbella E. Menicucci D. Neri B.. (2018). How breath-control can change your life: a systematic review on psycho-physiological correlates of slow breathing. Front. Hum. Neurosci. 12:353. doi: 10.3389/fnhum.2018.00353, PMID: 30245619 PMC6137615

[ref98] Zadkhosh S. M. Zandi H. G. Hemayattalab R. (2018). Neurofeedback versus mindfulness on young football players anxiety and performance. Turk. J. Kinesiol. 4, 132–141. doi: 10.31459/turkjkin.467470

[ref99] Zaichkowsky L. D. Fuchs C. Z. (1988). Biofeedback applications in exercise and athletic performance. Exerc. Sport Sci. Rev. 16, 381–422. 3292263

