# Supplementary material for: Self-regulation training improves stress resilience in elite pre-pubescent female gymnasts
Source: Front Psychol. 2024 Apr 24;15:1341437. doi: 10.3389/fpsyg.2024.1341437 (PMC11076701; doi:10.3389/fpsyg.2024.1341437)
Supplement: Supplementary file 1 [file Data_Sheet_1.docx]

Supplementary Material

This document serves as an extension to our main article, providing additional information on the analyses performed on both Biofeedback and Psychological metrics that were monitored and discussed therein. The purpose of this supplementary material is to delve deeper into the intricacies of our findings, offering a more comprehensive understanding of the data through descriptive analysis and the inclusion of additional figures. The analyses presented here aim to shed further light on the nuanced aspects of Physiological and Psychological metrics, which could not be fully explored within the main article due to space constraints. By providing this detailed extension, we aim to enrich the reader's comprehension of the core findings and facilitate a more robust discussion around the implications of our research. Readers are encouraged to refer to this supplementary document for a thorough exploration of the analyses and insights that support and expand upon the conclusions drawn in our primary study. The additional figures included are designed to provide a visual representation of the data, enhancing the interpretability and accessibility of our research findings.

# Detailed Data Pre-Processing Methodology for Physiological Data.

In our analysis, a meticulous approach was employed to process the physiological data. This was essential to accurately consider the intra-individual variations of physiological parameters both between (such as metabolism levels and within the evaluation sessions (including factors like hydration). The analysis was structured to distinctly capture the fluctuations in these parameters during both stress and recovery phases, categorized under the time variable.

*Normalization Process.* To facilitate a standardized comparison across participants, we applied min-max normalization to all physiological signals. This normalization process is critical for mitigating the impact of individual differences in baseline physiological states. By scaling the data within a range of 0 to 1, where the minimum and maximum values of each participant’s dataset are set as 0 and 1 respectively, we ensured that the subsequent analysis was based on relative changes rather than absolute values. This method effectively balances the data, making it comparable across individuals.

*Establishing Baseline Values.* The next step involved establishing a baseline value for each participant. This value is pivotal as it serves as a reference point for the subject’s resting physiological state. The baseline was recorded over a 4-minute period at the start of each session, as outlined in the methodological segment. To determine this baseline, we calculated the mean values of the physiological parameters during this resting period. These mean values represent the average physiological state of a subject when at rest, free from external stressors or recovery influences.

*Comparative Analysis with Stress and Recovery Phases*. Subsequently, these baseline values were utilized in a comparative analysis with the physiological responses recorded during the stress tasks and recovery periods within the assessment framework. Specifically, the mean baseline values were subtracted from the peak values observed for the same physiological parameters during the stress and recovery phases. This subtraction method allowed us to isolate the effects of stress and recovery activities on the physiological parameters. It provides a clear and normalized reference point against which changes can be precisely measured. This differential calculation highlights the extent to which physiological parameters deviate from the resting state under stress and how they return to baseline during recovery, offering valuable insights into the physiological dynamics of stress response and recovery processes.

In summary, this comprehensive data pre-processing approach ensures that our analysis of physiological data is both standardized across participants and sensitive to the intra-individual variations that occur in response to stress and recovery activities. The normalization and baseline establishment procedures are crucial for accurately interpreting the physiological impacts of the assessed activities.

# Physiological metrics - additional analysis.

## Skin Conductance Level.

**
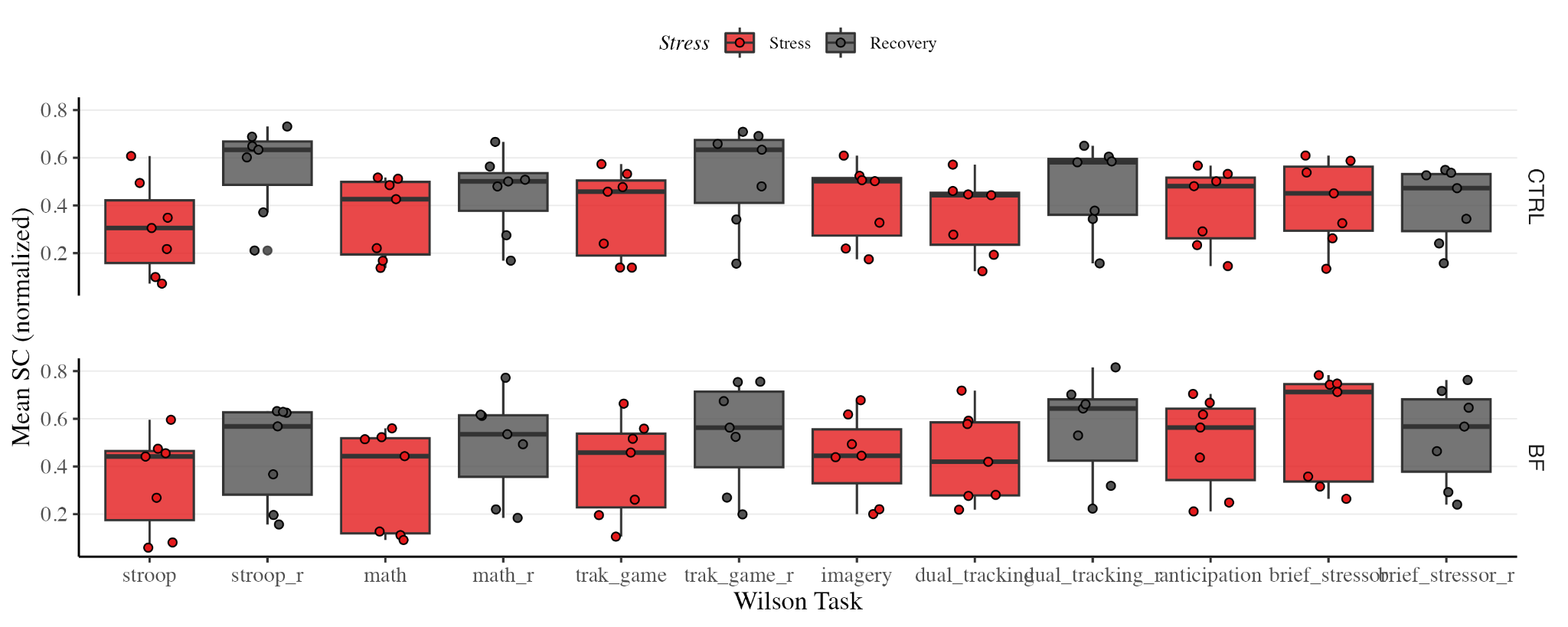
**

**Supplementary Figure 1.** Normalized Skin Conductance (on average, aggregate by Condition).

**
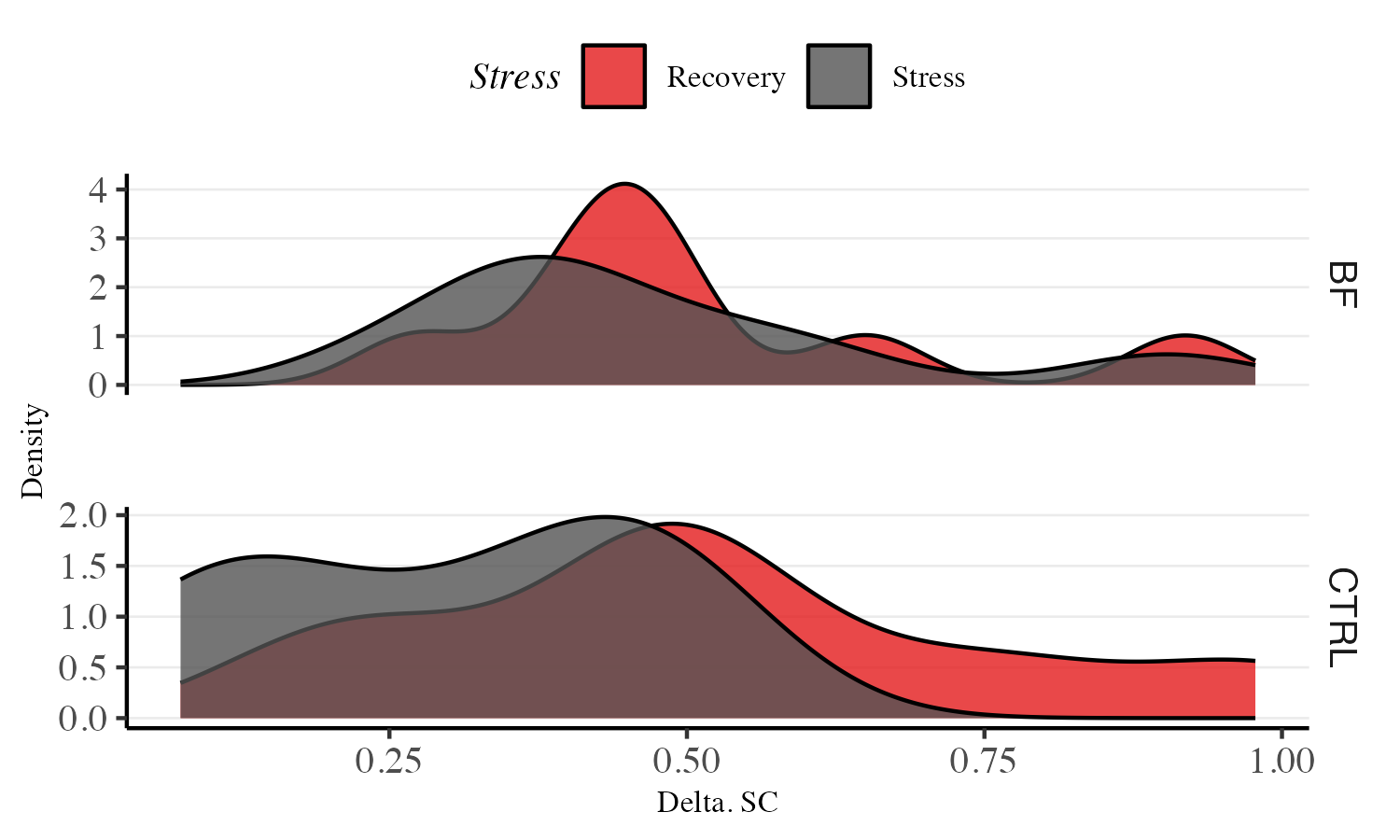
**

**Supplementary Figure 2.** Distribution of Delta Skin Conductance aggregate per condition (CTRL and BF * Stress and Recovery).


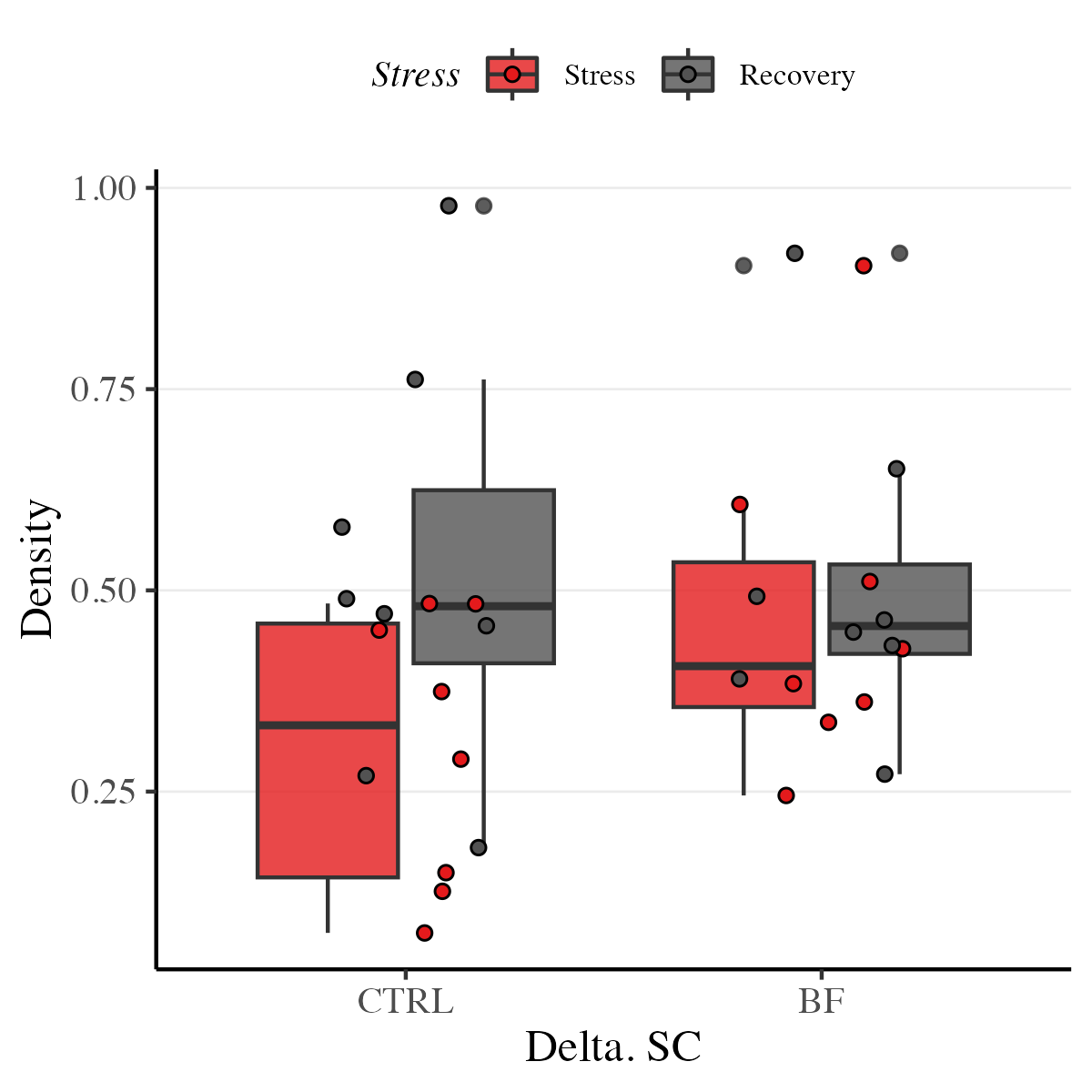


**Supplementary Figure 3**. Delta Skin Conductance aggregate by condition and stress for each participant.

**Supplementary Table 1.** Summary of raw and normalized data for Skin Conductance Levels.

|  | **CTRL** | | **BF** | |
| --- | --- | --- | --- | --- |
|  | **Recovery** | **Stress** | **Recovery** | **Stress** |
| SC *mean (sd)* | 8.46 (1.06) | 7.56 (0.89) | 9.2 (2.33) | 8.29 (2.24) |
| SC normalized *mean (sd)* | 0.42 (0.22) | 0.32 (0.19) | 0.49 (0.21) | 0.39 (0.19) |

**Supplementary Table 2.** Summary of Delta Skin Conductance and Assumptions checks for the 2x2 repeated measures ANOVA.

|  | **CTRL** | | **BF** | |
| --- | --- | --- | --- | --- |
|  | **Recovery** | **Stress** | **Recovery** | **Stress** |
| Shapiro-Wilk | 0.950 | 0.964 | 0.832 | 0.921 |
| P-value of Shapiro-Wilk | 0.713 | 0.851 | 0.063 | 0.435 |
| Mean (sd) | 0.52 (0.26) | 0.3 (0.17) | 0.51 (0.2) | 0.47 (0.21) |

**Supplementary Table 3.** Simple Main Effects for the 2x2 repeated measures ANOVA on Skin Conductance.

|  | **Level** | **Sum of Squares** | **df** | **Mean Square** | **F** | **p** |
| --- | --- | --- | --- | --- | --- | --- |
| *Condition* | Stress | 0.166 | 1 | 0.166 | 7.750 | 0.027 |
|  | Recovery | 0.029 | 1 | 0.029 | 0.645 | 0.448 |
|  | CTRL | 0.145 | 1 | 0.145 | 12.295 | 0.010 |
| *Time* | BF | 0.020 | 1 | 0.020 | 6.854 | 0.035 |

## Peripheral Temperature.


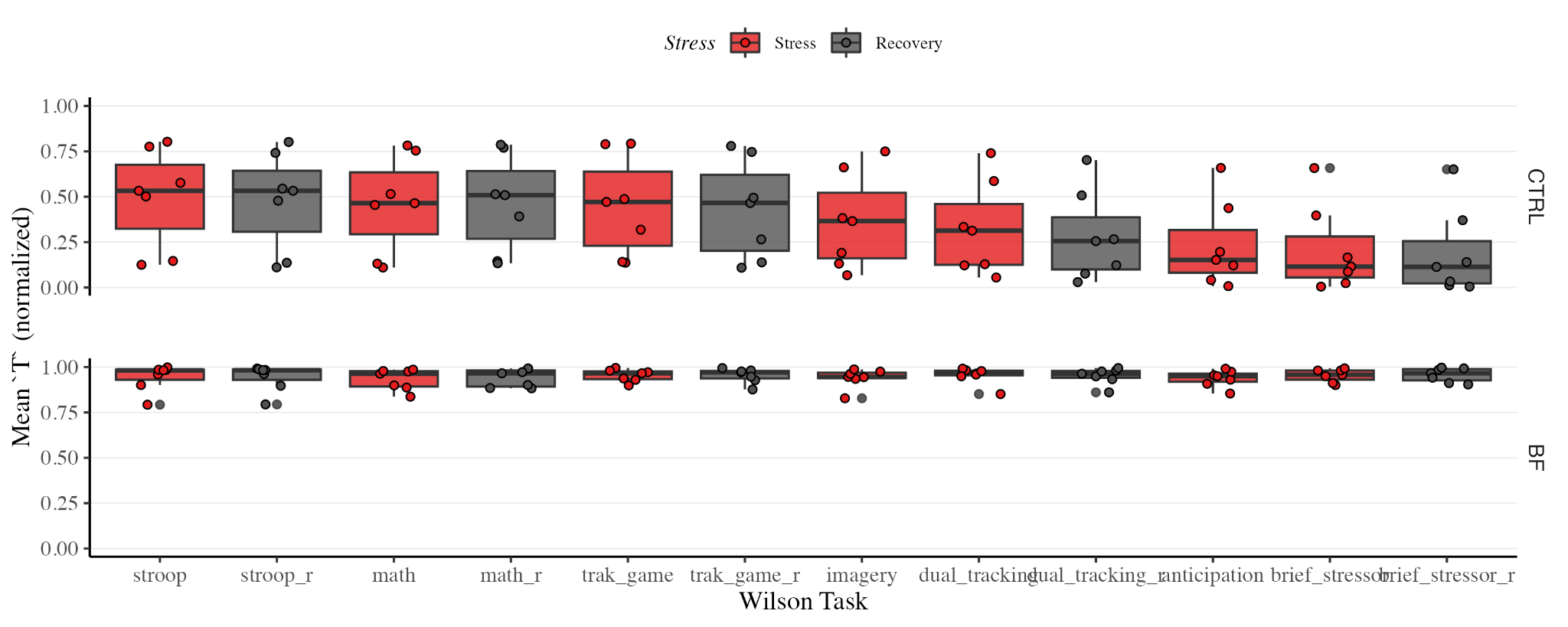


**Supplementary Figure 4.** Normalized Temperature (on average, aggregate by Condition).


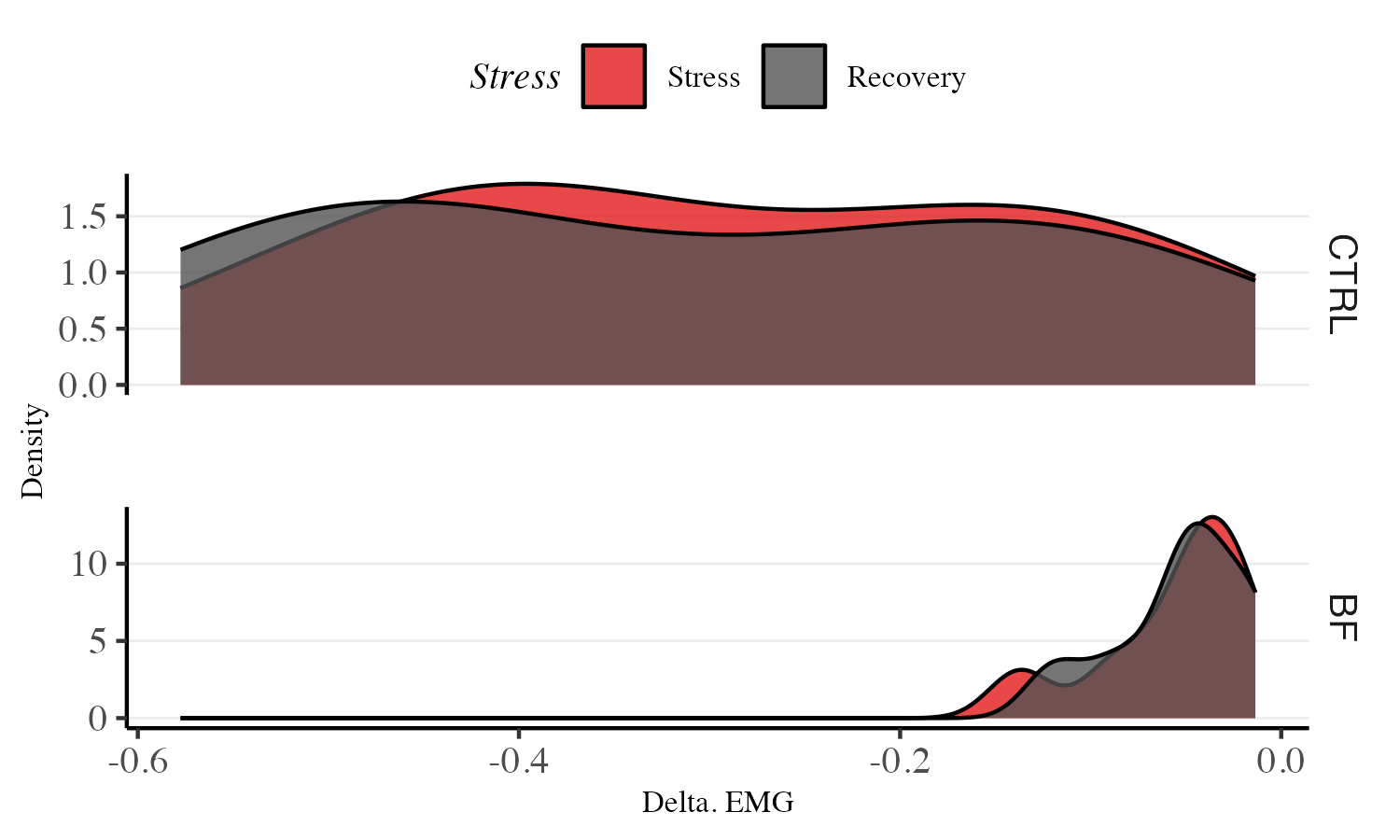


**Supplementary Figure 5**. Distribution of Delta Temperature aggregate per condition (CTRL and BF * Stress and Recovery).


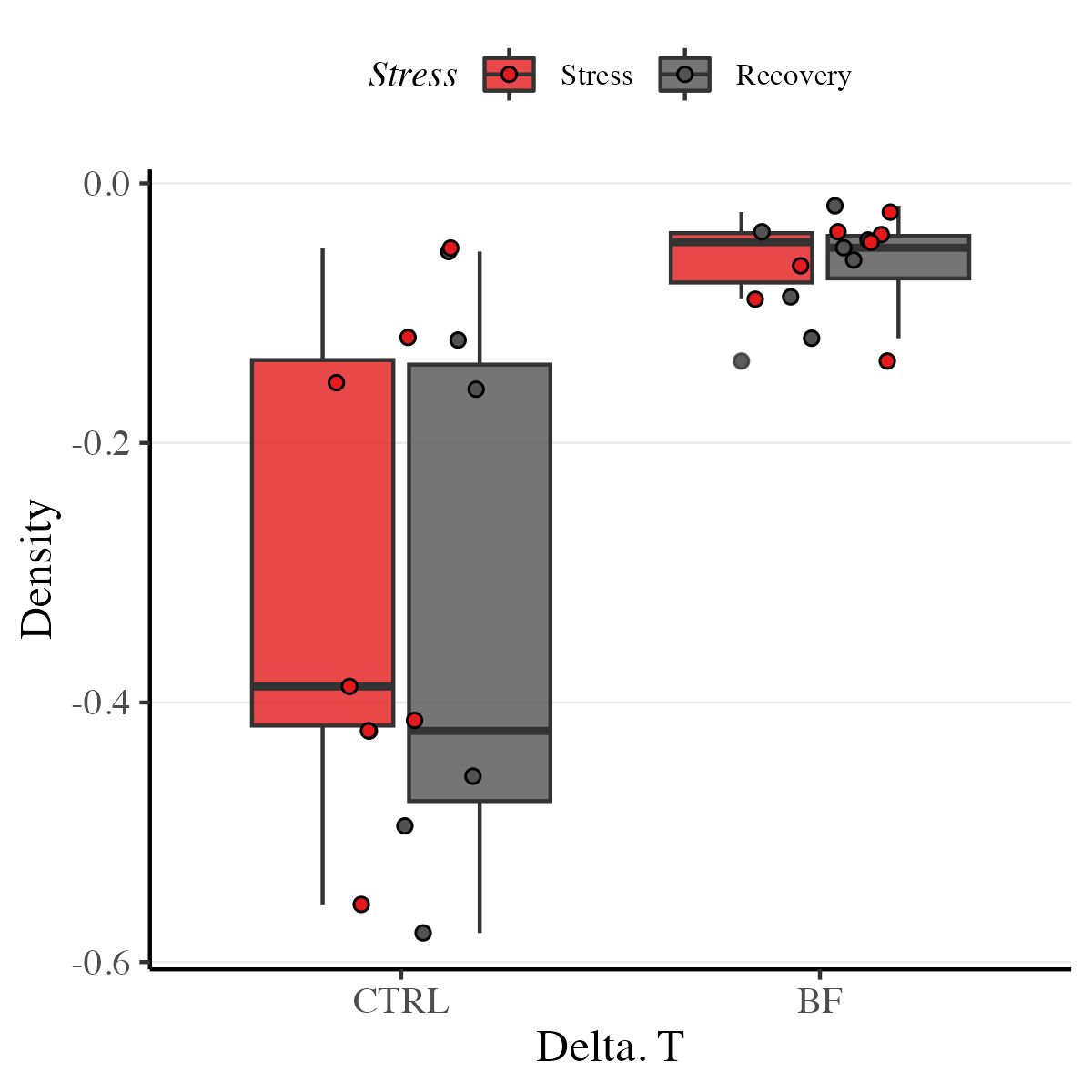


**Supplementary Figure 6.** Delta Temperature aggregate by condition and stress for each participant.

**Supplementary Table 4.** Summary of pre and normalized for Temperature.

|  | **CTRL** | | **BF** | |
| --- | --- | --- | --- | --- |
|  | **Recovery** | **Stress** | **Recovery** | **Stress** |
| T *mean (sd)* | 26.95 (3.59) | 27.26 (3.66) | 34.26 (1.43) | 34.20 (1.47) |
| T normalized *mean (sd)* | 0.35 (0.26) | 0.38 (0.25) | 0.94 (0.04) | 0.94 (0.05) |

**Supplementary Table 5.** Summary of Delta Temperature and Assumptions checks for the 2x2 repeated measures ANOVA.

|  | **CTRL** | | **BF** | |
| --- | --- | --- | --- | --- |
|  | **Recovery** | **Stress** | **Recovery** | **Stress** |
| Shapiro-Wilk | 0.928 | 0.944 | 0.917 | 0.880 |
| P-value of Shapiro-Wilk | 0.496 | 0.651 | 0.405 | 0.189 |
| Mean (sd) | -0.32 (0.19) | -0.29 (0.18) | -0.05 (0.04) | -0.05 (0.05) |

**Supplementary Table 6.** Simple Main Effects for the 2x2 repeated measures ANOVA on Temperature.

|  | **Level of Time** | **Sum of Squares** | **df** | **Mean Square** | **F** | **p** |
| --- | --- | --- | --- | --- | --- | --- |
| *Condition* | Stress | 0.225 | 1 | 0.225 | 17.469 | 0.004 |
|  | Recovery | 0.285 | 1 | 0.285 | 17.354 | 0.004 |
|  | CTRL | 0.003 | 1 | 0.003 | 7.148 | 0.032 |
| *Time* | BF | 8.595×10^-5^ | 1 | 8.595×10^-5^ | 2.164 | 0.185 |

## EMG - muscle activity.


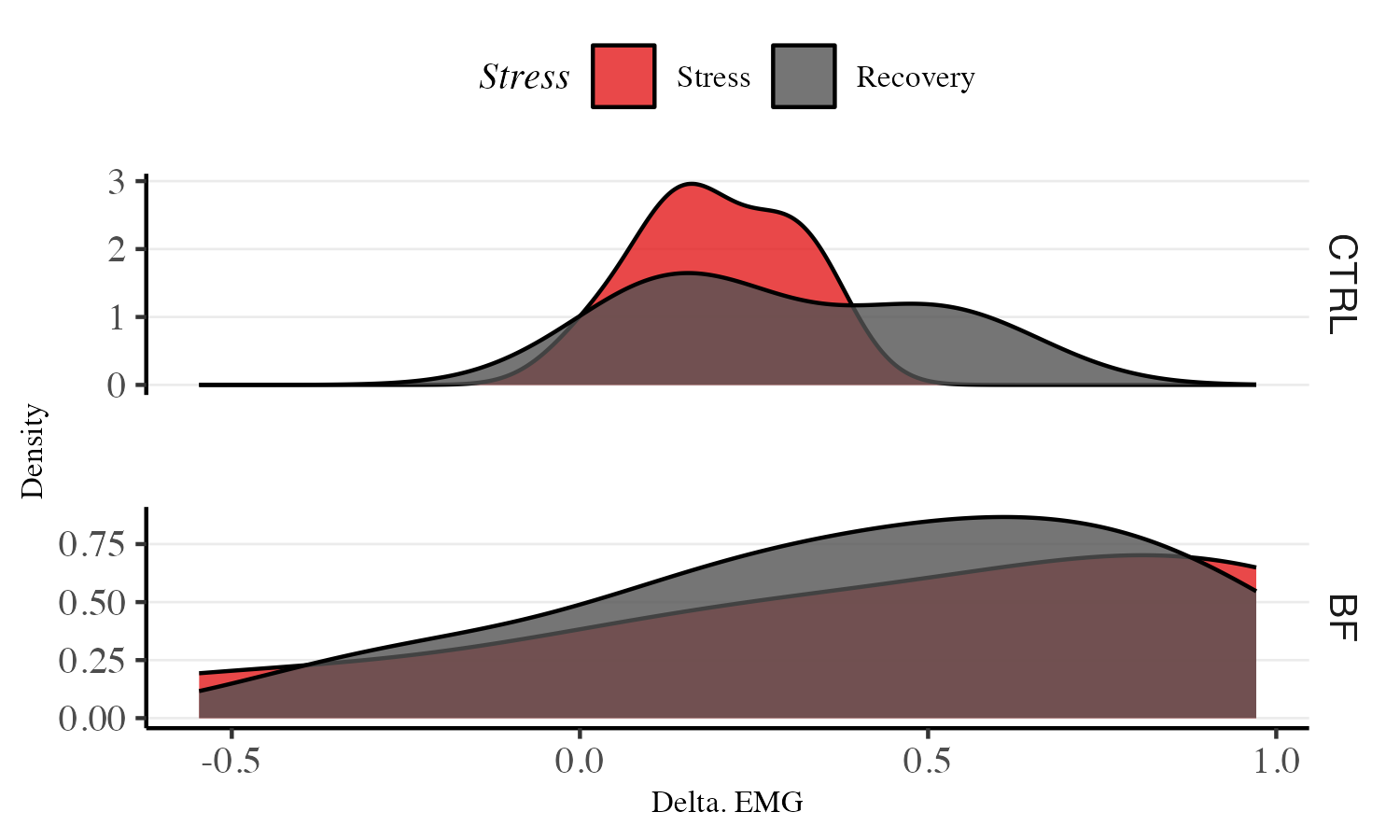


**Supplementary Figure 7.** Distribution of Delta EMG aggregate per condition (CTRL and BF * Stress and Recovery).


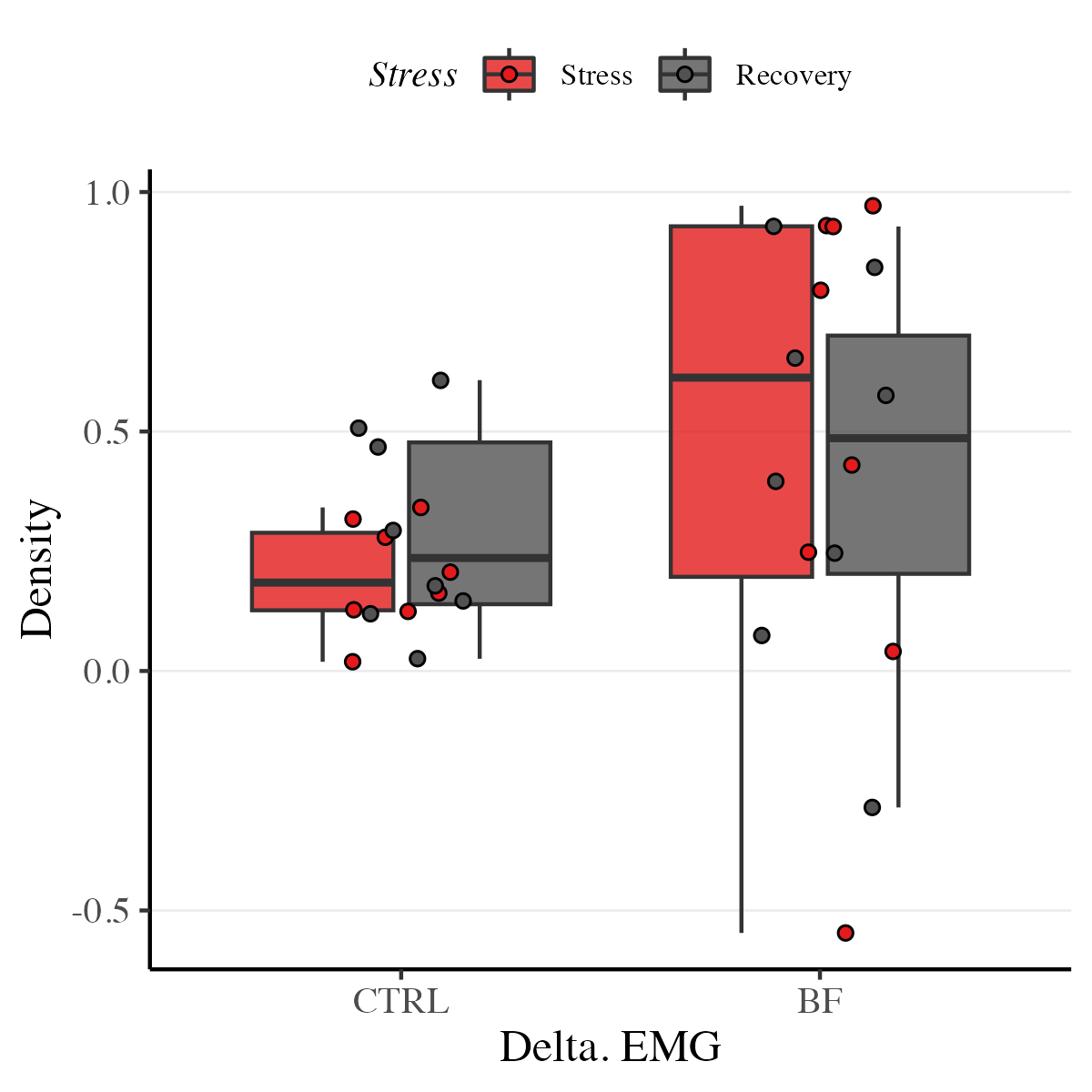


**Supplementary Figure 8.** Delta EMG aggregate by condition and stress for each participant.

**Supplementary Table 7.** Summary of pre and normalized for EMG.

|  | **CTRL** | | **BF** | |
| --- | --- | --- | --- | --- |
|  | **Recovery** | **Stress** | **Recovery** | **Stress** |
| EMG *mean (sd)* | 14.28 (7.71) | 8.41 (5.07) | 22.53 (29.67) | 28.65 (64.99) |
| EMG normalized *mean (sd)* | 0.03 (0.02) | 0.01 (0.01) | 0.04 (0.05) | 0.05 (0.1) |

**Supplementary Table 8.**  Summary of Delta EMG and Assumptions checks for the 2x2 repeated measures ANOVA.

|  | **CTRL** | | **BF** | |
| --- | --- | --- | --- | --- |
|  | **Recovery** | **Stress** | **Recovery** | **Stress** |
| Shapiro-Wilk | 0.927 | 0.955 | 0.964 | 0.876 |
| P-value of Shapiro-Wilk | 0.492 | 0.765 | 0.847 | 0.174 |
| Mean (sd) | 0.29 (0.21) | 0.2 (0.11) | 0.43 (0.41) | 0.47 (0.54) |

**Supplementary Table 9.** Simple Main Effects for the 2x2 repeated measures ANOVA on EMG.

|  | **Level of Time** | **Sum of Squares** | **df** | **Mean Square** | **F** | **p** |
| --- | --- | --- | --- | --- | --- | --- |
| *Condition* | Stress | 0.308 | 1 | 0.308 | 2.311 | 0.172 |
|  | Recovery | 0.074 | 1 | 0.074 | 0.938 | 0.365 |
|  | CTRL | 0.037 | 1 | 0.037 | 4.969 | 0.061 |
| *Time* | BF | 0.008 | 1 | 0.008 | 0.156 | 0.705 |

## Blood Volume Pressure.


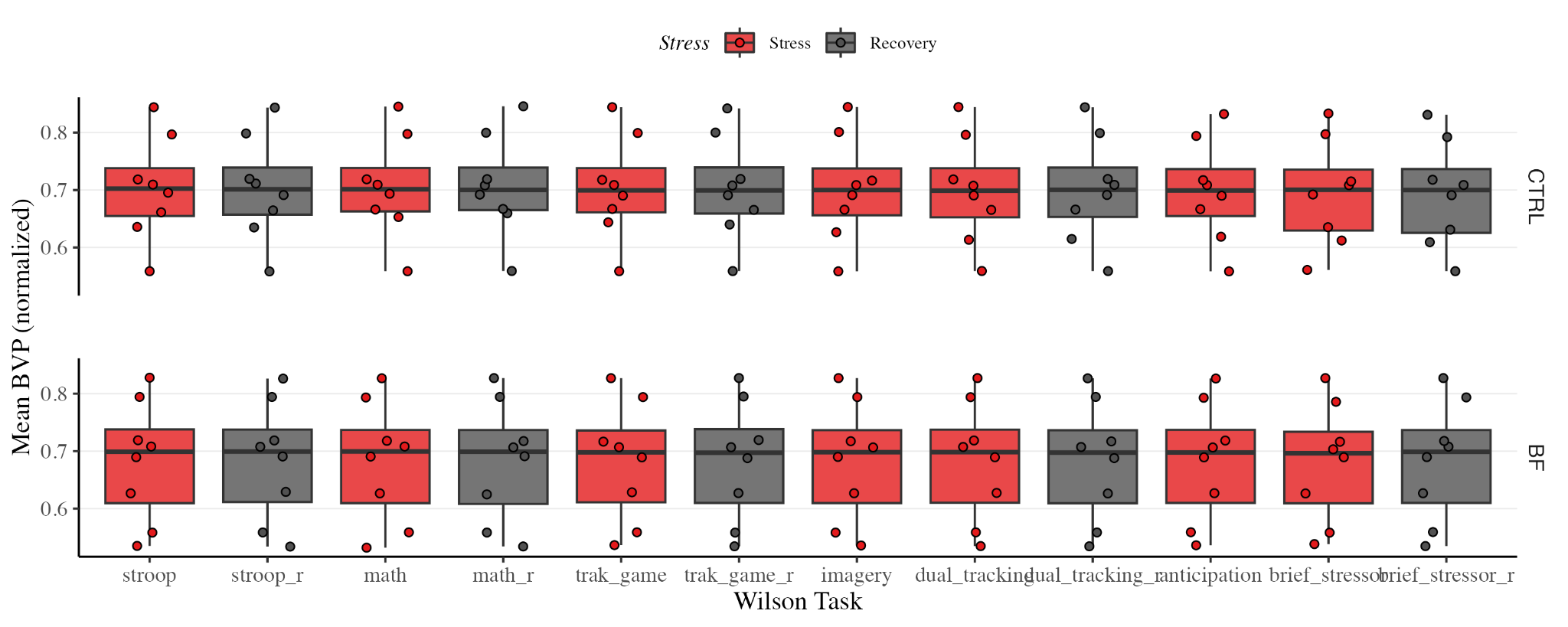


**Supplementary Figure 9.** Normalized BVP (on average, aggregate by Condition).


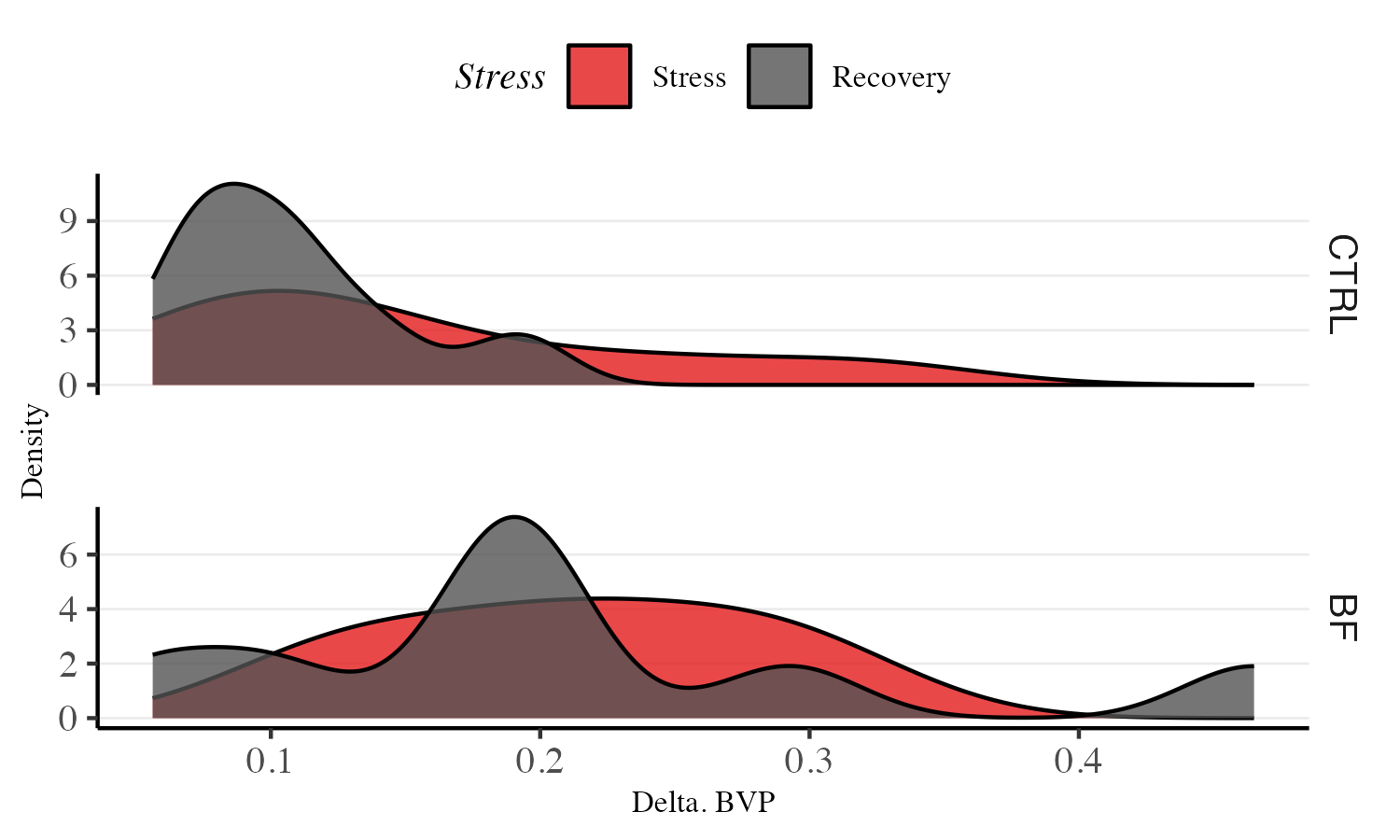


**Supplementary Figure 10.** Distribution of Delta BVP aggregate per condition (CTRL and BF * Stress and Recovery).


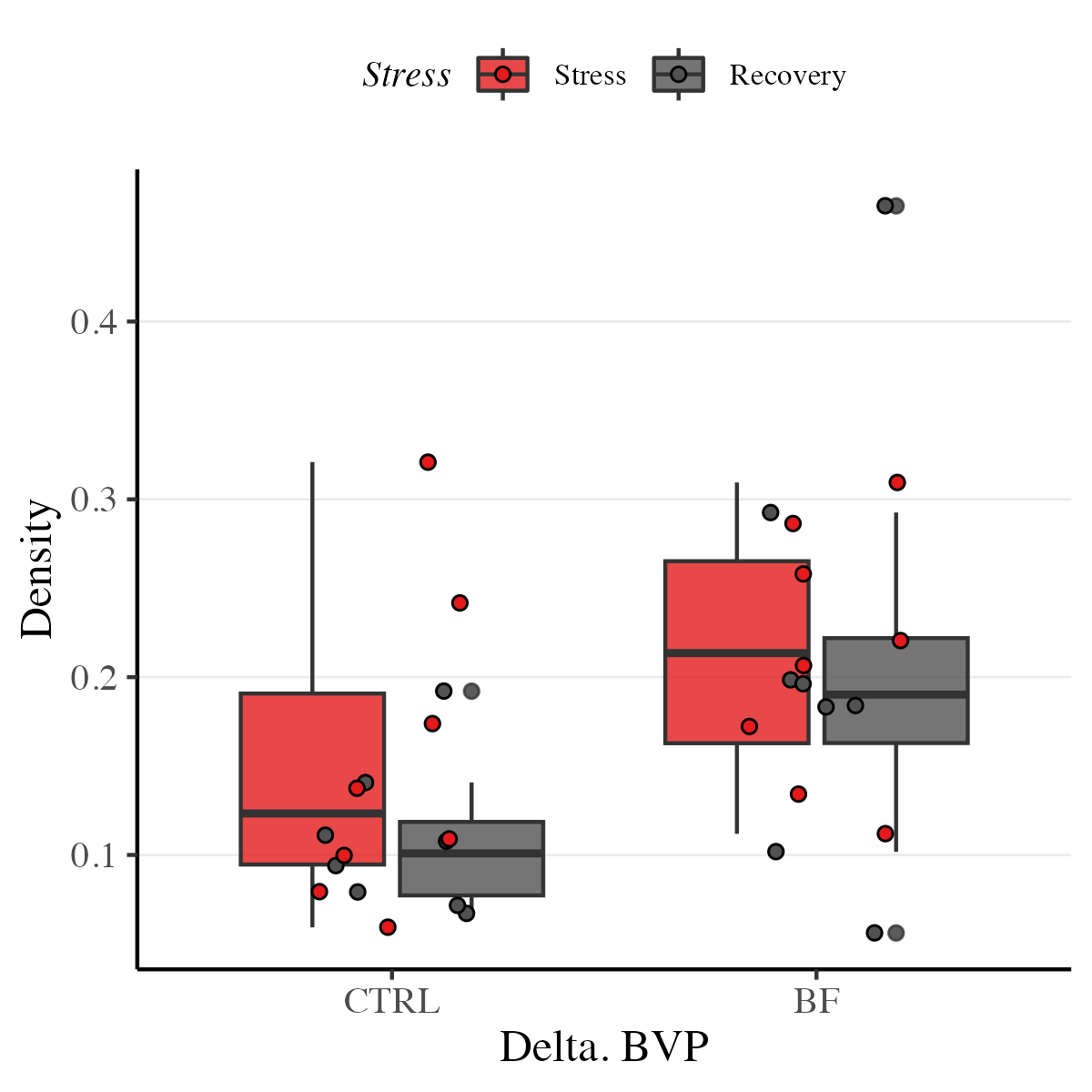


**Supplementary Figure 11**. Delta BVP aggregate by condition and stress for each participant.

**Supplementary Table 10.** Summary of pre and normalized for BVP.

|  | **CTRL** | | **BF** | |
| --- | --- | --- | --- | --- |
|  | **Recovery** | **Stress** | **Recovery** | **Stress** |
| BVP *mean (sd)* | 38.12 (2.53) | 38.18 (2.59) | 36.67 (0.03) | 36.69 (0.02) |
| BVP normalized *mean (sd)* | 0.7 (0.09) | 0.7 (0.09) | 0.68 (0.1) | 0.68 (0.1) |

**Supplementary Table 11.**  Summary of Delta BVP and Assumptions checks for the 2x2 repeated measures ANOVA.

|  | **CTRL** | | **BF** | |
| --- | --- | --- | --- | --- |
|  | **Recovery** | **Stress** | **Recovery** | **Stress** |
| Shapiro-Wilk | 0.859 | 0.996 | 0.965 | 0.847 |
| P-value of Shapiro-Wilk | 0.118 | 1.000 | 0.859 | 0.089 |
| Mean (sd) | 0.11 (0.04) | 0.15 (0.09) | 0.21 (0.12) | 0.21 (0.07) |

**Supplementary Table 12.** Simple Main Effects for the 2x2 repeated measures ANOVA on BVP.

|  | **Level of Time** | **Sum of Squares** | **df** | **Mean Square** | **F** | **p** |
| --- | --- | --- | --- | --- | --- | --- |
| *Condition* | Stress | 0.014 | 1 | 0.014 | 2.065 | 0.194 |
|  | Recovery | 0.041 | 1 | 0.041 | 7.293 | 0.031 |
|  | CTRL | 0.008 | 2.983 | 0.128 | 0.008 | 2.983 |
| *Time* | BF | 3.030×10-5 | 0.004 | 0.954 | 3.030×10^-5^ | 0.004 |

## Respiration Rate.

**
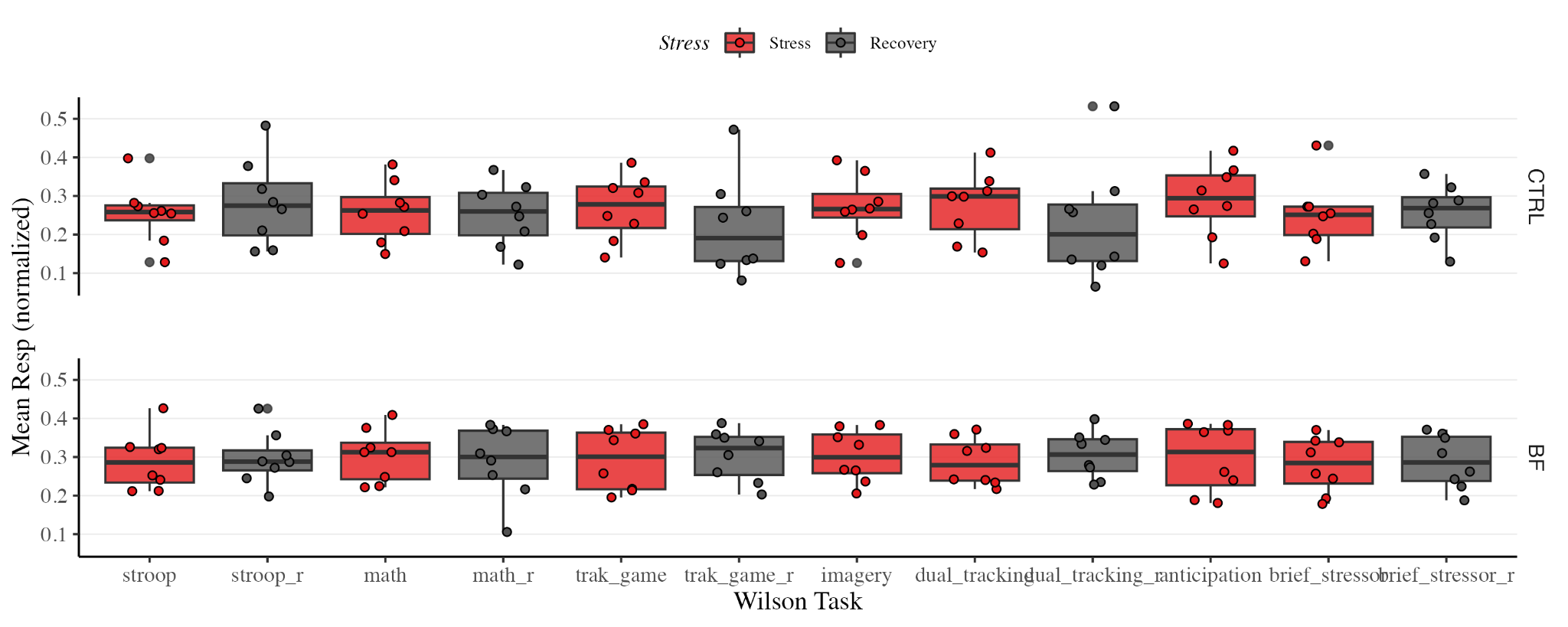
**

**Supplementary Figure 12.** Normalized Respiration (on average, aggregate by Condition).


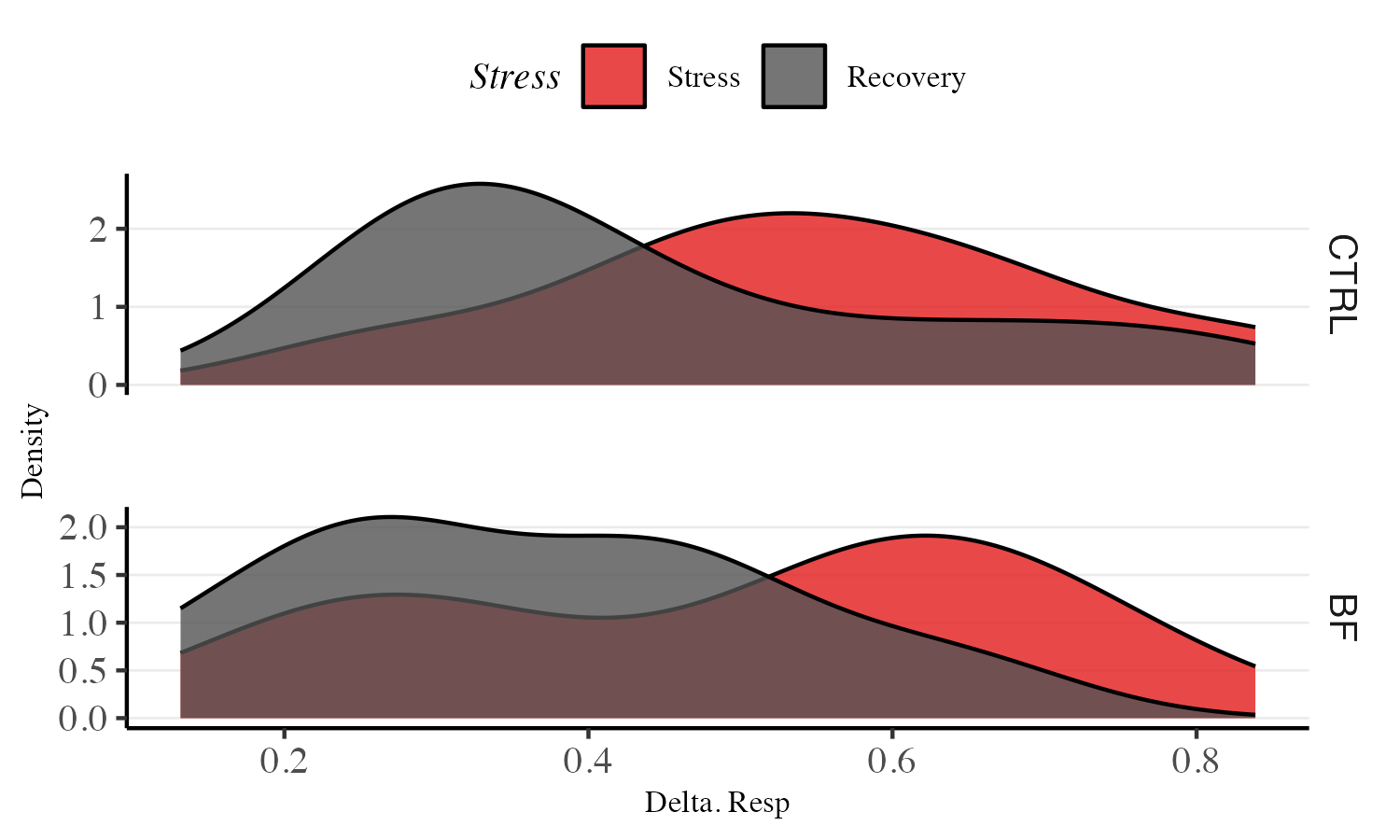


**Supplementary Figure 13.** Distribution of Delta Respiration aggregate per condition (CTRL and BF * Stress and Recovery).


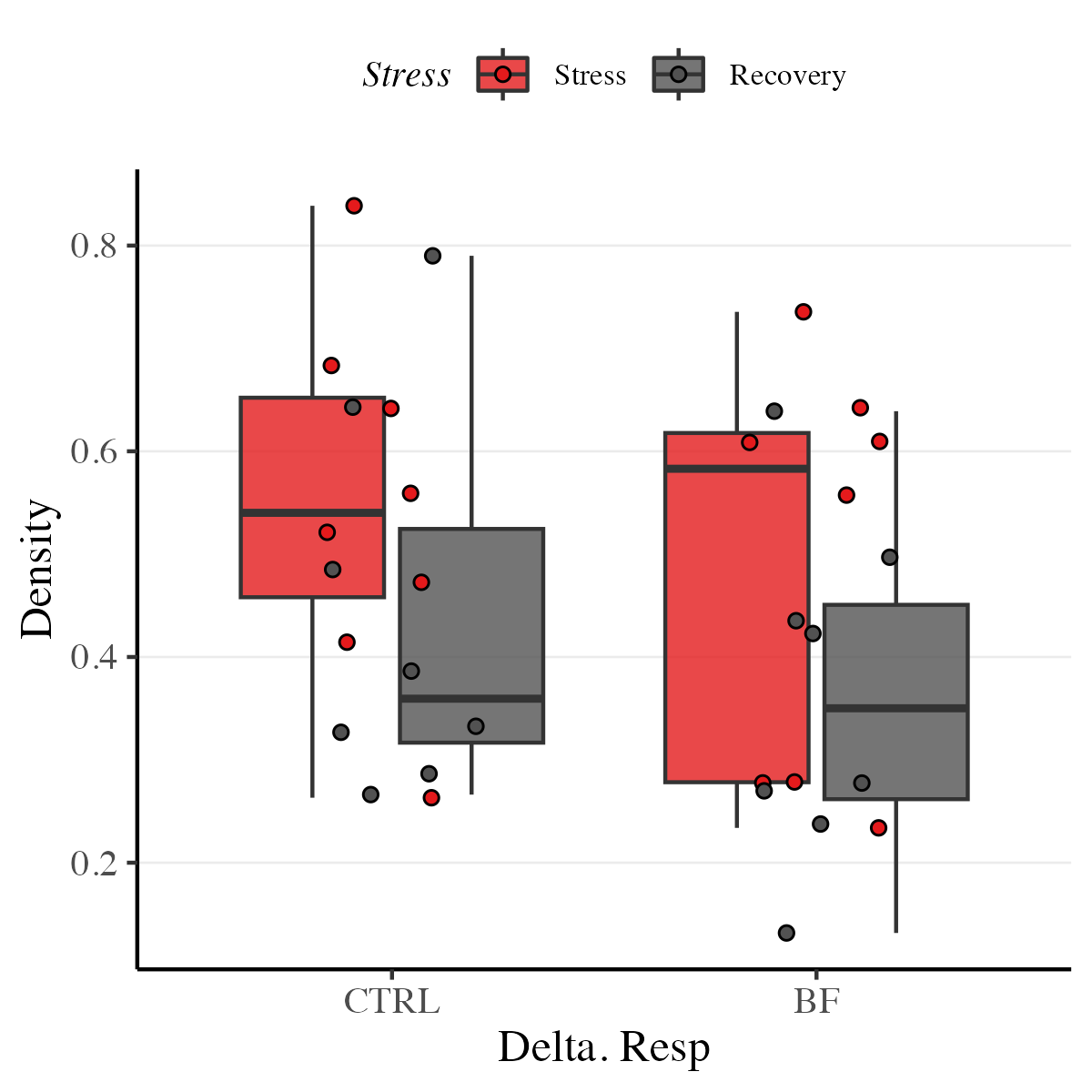


**Supplementary Figure 14.** Delta Respiration aggregate by condition and stress for each participant.

**Supplementary Table 13.** Summary of pre and normalized for Respiration.

|  | **CTRL** | | **BF** | |
| --- | --- | --- | --- | --- |
|  | **Recovery** | **Stress** | **Recovery** | **Stress** |
| Respiration *mean (sd)* | 35.41 (2.99) | 35.94 (2.63) | 37 (3.54) | 36.98 (3.84) |
| Respiration normalized *mean (sd)* | 0.24 (0.09) | 0.27 (0.08) | 0.3 (0.06) | 0.29 (0.07) |

**Supplementary Table 14.** Summary of Delta Respiration and Assumptions checks for the 2x2 repeated measures ANOVA.

|  | **CTRL** | | **BF** | |
| --- | --- | --- | --- | --- |
|  | **Recovery** | **Stress** | **Recovery** | **Stress** |
| Shapiro-Wilk | 0.859 | 0.996 | 0.965 | 0.847 |
| P-value of Shapiro-Wilk | 0.118 | 1.000 | 0.859 | 0.089 |
| Mean (sd) | 0.44 (0.19) | 0.55 (0.18) | 0.36 (0.16) | 0.49 (0.2) |

**Supplementary Table 15.** Simple Main Effects for the 2x2 repeated measures ANOVA on Respiration.

|  | **Level of Time** | **Sum of Squares** | **df** | **Mean Square** | **F** | **p** |
| --- | --- | --- | --- | --- | --- | --- |
| *Condition* | Stress | 0.013 | 1 | 0.013 | 0.243 | 0.637 |
|  | Recovery | 0.023 | 1 | 0.023 | 0.602 | 0.463 |
|  | CTRL | 0.048 | 1 | 0.048 | 1.403 | 0.275 |
| *Time* | BF | 0.067 | 1 | 0.067 | 5.085 | 0.059 |

# Additional metrics and visualization.

**
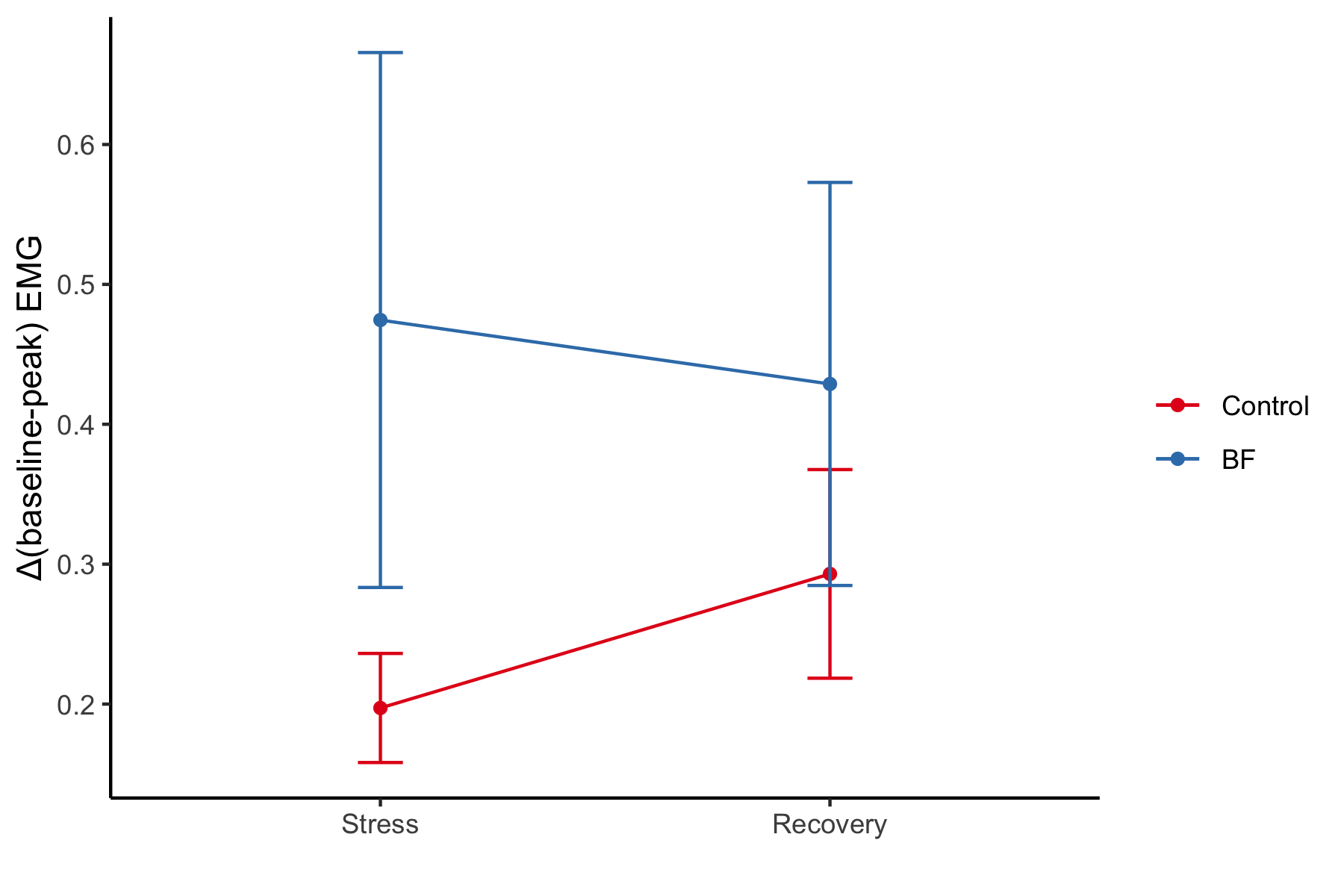
**

**Supplementary Figure 16.** The effect of treatment on Muscle Activity. Image shows the effects of treatment on EMG in BF (blue) and CTRL (red) conditions during baseline stress, and recovery phases. Vertical bars represent standard errors.

*
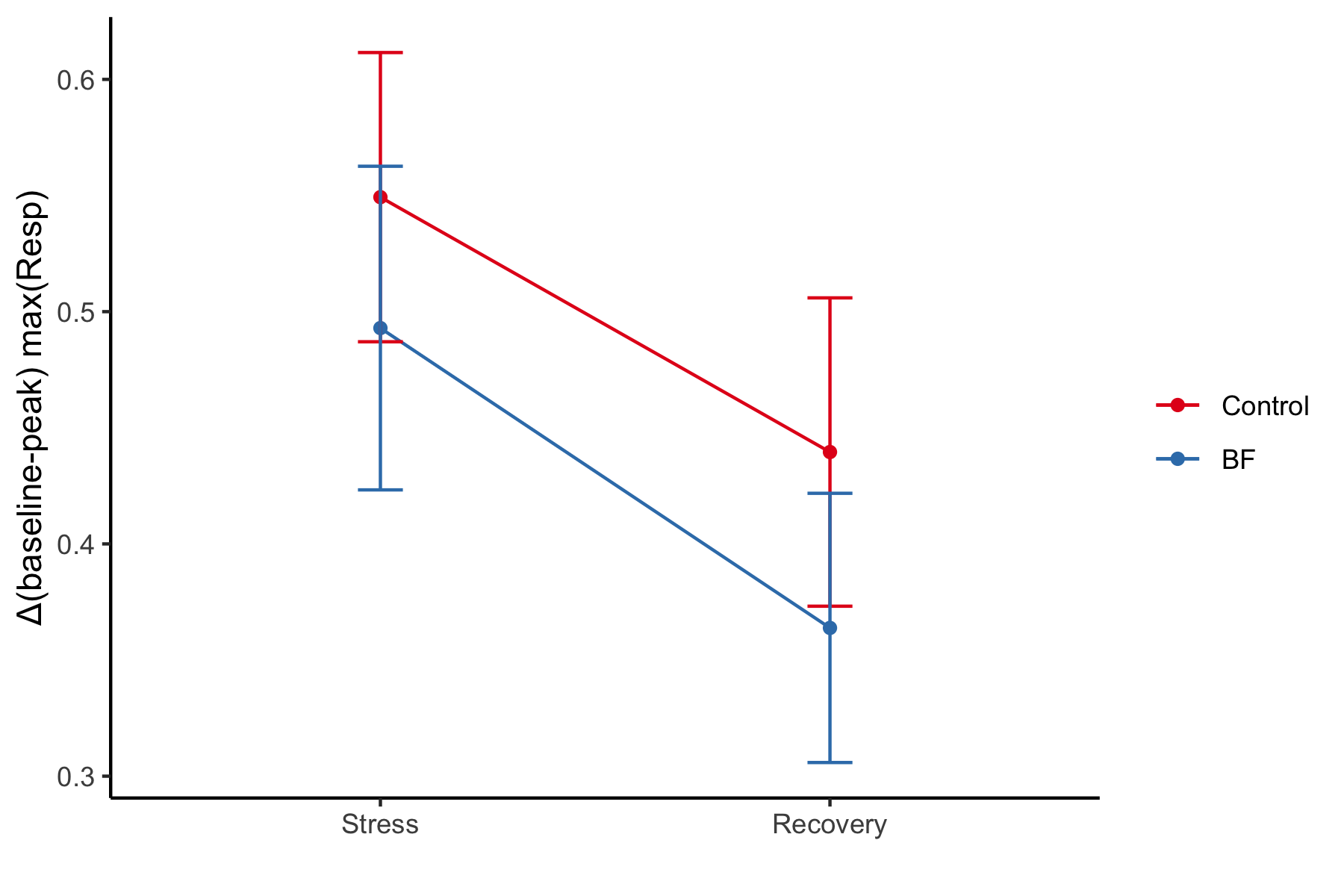
*

**Supplementary Figure 17.** The effect of treatment on Respiration rate. Image shows the effects of treatment on Respiration in BF (blue) and CTRL (red) conditions during baseline stress, and recovery phases. Vertical bars represent standard errors.

**
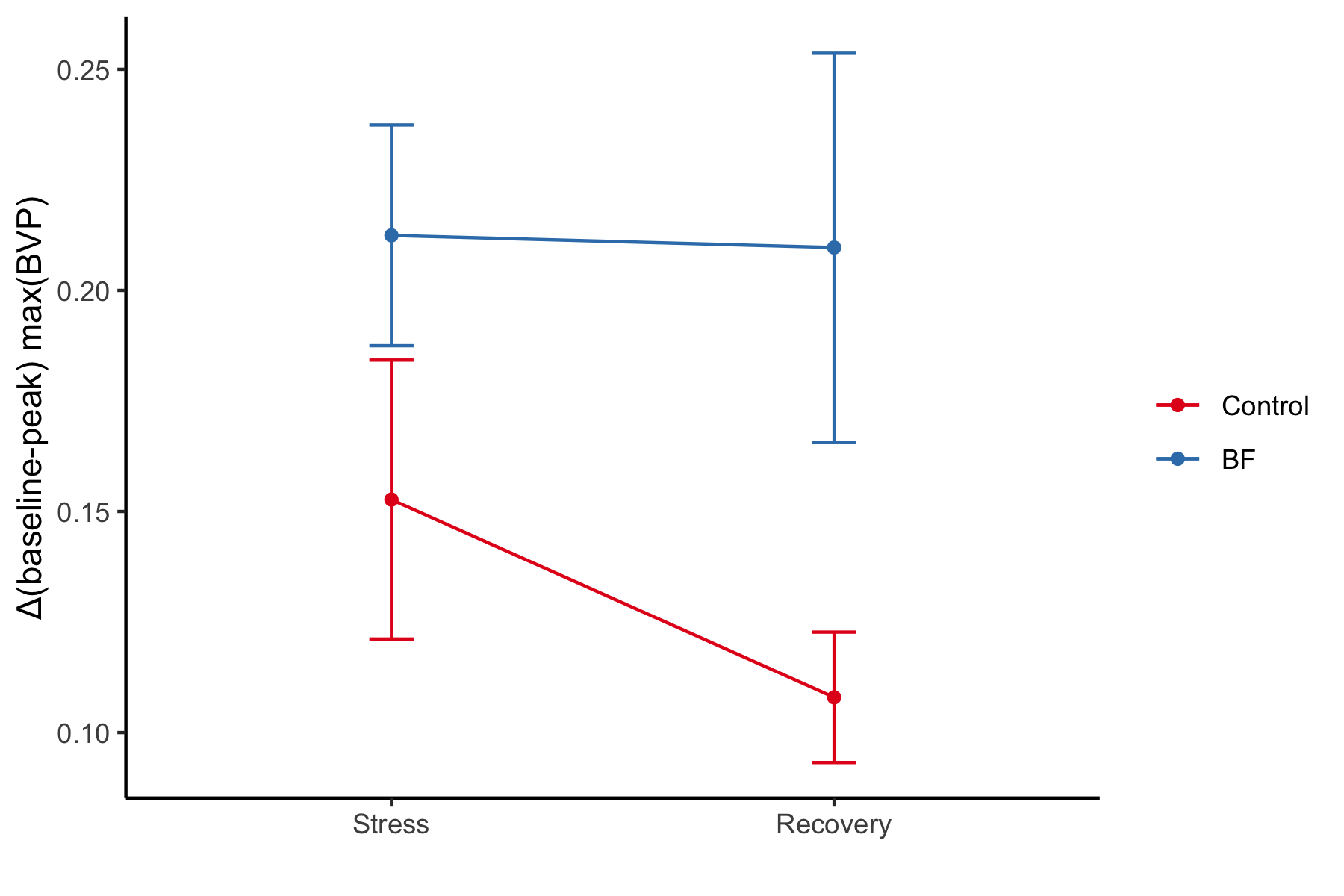
**

**Supplementary Figure 18.** The effect of treatment on Blood Volume Pressure. Image shows the effects of treatment on BVP in BF (blue) and CTRL (red) conditions during baseline stress, and recovery phases. Vertical bars represent standard errors.

# Psychological metrics - additional analysis.

The analysis of psychological data took into consideration the inherent variations within each subject. For a comprehensive understanding, averages were meticulously calculated based on the results obtained from the administered questionnaires, both prior to and following each distinct experimental condition, namely, the control condition (CTRL) and the biofeedback condition (BF).

**Supplementary Table 16.** Assumptions Check.

| **MAIA subscale** |  | **Mean (sd)** | **Shapiro-Wilk** | **P-value of Shapiro-Wilk** |
| --- | --- | --- | --- | --- |
| MAIA Total Score | CTRL | 72.62 (13.65) | 0.94 | 0.66 |
|  | BF | 93.25 (19.62) | 0.94 | 0.60 |
| Noticing | CTRL | 2.37 (0.60) | 0.89 | 2.24 |
|  | BF | 2.81 (0.72) | 0.87 | 0.16 |
| Not Distracting | CTRL | 1.87 (0.85) | 0.84 | 0.07 |
|  | BF | 2.96 (0.96) | 0.93 | 0.52 |
| Not Worrying | CTRL | 2.62 (0.93) | 0.85 | 0.10 |
|  | BF | 2.79 (1.25) | 0.95 | 0.68 |
| Attention Regulation | CTRL | 1.71 (0.77) | 0.82 | 0.05 |
|  | BF | 2.50 (0.87) | 0.96 | 0.81 |
| Emotional Awareness | CTRL | 2.63 (0.73) | 0.92 | 0.44 |
|  | BF | 3.40 (0.71) | 0.98 | 0.98 |
| Self-regulation | CTRL | 2.19 (1.05) | 0.84 | 0.08 |
|  | BF | 3.09 (0.61) | 0.88 | 0.20 |
| Body Listening | CTRL | 2.00 (0.87) | 0.92 | 0.40 |
|  | BF | 2.38 (1.05) | 0.92 | 0.47 |
| Trusting | CTRL | 3.25 (1.16) | 0.83 | 0.06 |
|  | BF | 3.58 (1.24) | 0.85 | 0.09 |
